# Mechanical Force Storage and Reprogramming Hydrogel for Scarless Repair of Sports Joint Wounds

**DOI:** 10.1002/advs.202511332

**Published:** 2025-09-30

**Authors:** Jiakang Zhang, Yuhui Zhang, Yuanru Lang, Tao Fu, Qian Liu, Long Chen, Peng Zhang, Yan Xiao, Yuntong Zhang, Shuo Fang, Meidong Lang

**Affiliations:** ^1^ Shanghai Key Laboratory of Advanced Polymeric Materials Key Laboratory for Ultrafine Materials of Ministry of Education School of Materials Science and Engineering East China University of Science and Technology 130 Meilong Road Shanghai 200237 P. R. China; ^2^ Institute of Molecular Medicine Renji Hospital School of Medicine Shanghai Jiao Tong University Shanghai 200127 P. R. China; ^3^ Department of Trauma and Orthopedic Surgery The First Affiliated Hospital of Navy Medical University Shanghai 200433 P. R. China; ^4^ Department of Plastic Surgery The First Affiliated Hospital of Navy Medical University Shanghai 200433 P. R. China

**Keywords:** elastic recoil capacity, mechanical force storage and reprogramming hydrogels, mechanical signal transduction, sports wounds, wound margin stress

## Abstract

The wounds at the joints are subject to repeated pulling. This not only causes repeated rupture and bleeding of new granulation tissue, but also causes excessive exogenous mechanical stimulation of cell populations, leading to excessive cell proliferation and vascular proliferation at the trauma site. Inspired by the energy conversion of tendons, the “energy transit station” hydrogel is designed. When applied to dynamic joint wounds, the rigid cross‐linked network of hydrogels rapidly absorbs wound edge stress by elastic deformation and stores them as elastic potential energy in the topological network matrix, driving the hydrogels to exhibit programmable elastic recoil capabilities. Thus, the “energy transfer station” hydrogel not only shields stress concentration in sports injuries, but also reprograms energy forms to provide reasonable biomimetic contraction for wounds. In vivo research, compared with the control group (83.06%), this hydrogel can significantly accelerate the healing process of sports injuries (99.87%). The “energy transit station” property significantly downregulated the En1 lineage‐positive fibroblast population (only 9.21% of the control group) and coordinated the activation of α‐SMA‐positive myofibroblasts (only 14.62% of the control group). This research provides an innovative strategy for high‐quality healing of joint wounds through the conversion and re‐transmission of energy.

## Graphical abstract

Inspired by the energy conversion of tendons, the “energy transit station” hydrogel is designed. The hydrogel not only shields stress concentration in sports injuries, but also reprograms energy forms to provide reasonable biomimetic contraction for wounds. This research provides an innovative strategy for high‐quality healing of joint wounds through the conversion and re‐transmission of energy.

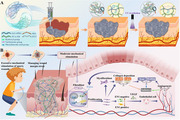

## 1 Introduction

Acute wounds frequently deteriorate into chronic wounds that are difficult to heal.^[^ ^1^ ^]^ This phenomenon can be attributed to various unfavorable factors, including organismal immune dysfunction, oxidative stress microenvironment, and bacterial infection.^[^ ^2^ ^]^ In recent decades, overreliance or inappropriate use of antibiotics has contributed to the evolution of bacteria into multidrug resistant bacteria.^[^ ^3^ ^]^ It has led to drug‐resistant bacterial infections and the formation of biofilms, which have become significant threats to open trauma patients globally.^[^ ^4^ ^]^ The uncertainty of trauma occurrence results in wounds inevitably occurring on the skin at the site of joint movement.^[^ ^5^ ^]^ This requires dressings that not only eliminate bacteria and biofilms already present in wounds, but also build infection defense lines for wounds continuously.^[^ ^6^ ^]^ Concurrently, dressings have the capacity to program marginal stresses in sports wounds, effectively managing the transmission of mechanical stress signals in wounds to prevent the formation of hyperplastic scars with misarranged collagen fibers, fibroblast activation, and abundant vascular distribution. Based on investigation, there is currently no intelligent dressing that can meet the needs of chronically infected sports wounds. Therefore, it is urgent to develop intelligent dressings that can both efficiently eliminate bacteria and biofilms to harmonize the inflammatory cascade response in chronic wounds, and effectively manage mechanical stresses on sports wounds to prevent them from entering into abnormal proliferation‐remodeling procedures.

Wound margin cells are capable of sensing mechanical forces and converting them into complex biochemical signals, thereby triggering corresponding signaling cascade reactions that coordinate the behavioral activities of various types of cells.^[^ ^7^ ^]^ Appropriate mechanical stimulation has positive effects on wound healing.^[^ ^8^ ^]^ The skin of joints is frequently subjected to substantial pulling and twisting due to the normal life activities of the body.^[^ ^9^ ^]^ Once the skin is injured in this area, the wound cells undergo excessive mechanical stimulation.^[^ ^10^ ^]^ It disrupts the normal repair procedures of the wound, particularly during the inflammatory and proliferative phases. In the inflammatory phase, frequent pulling exerts stress concentrations at the margins of wounds, resulting in the violent destruction of newly formed nascent granulation tissue. The rebleeding of tissues and repeated spillage of exudates may lead to the colonization and growth of drug‐resistant bacteria in the wound. This phenomenon may cause overstimulation of the immune system to recruit inflammatory cells and secrete inflammatory factors, resulting in severe infiltration of inflammatory cells and inflammatory factors in the wound. Additionally, this may hinder collagen deposition, fibroblast migration, and angiogenesis. Consequently, the wound enters into the vicious circle of inflammatory cascade reaction, thus the lesions remain in the inflammatory stage for long periods of time.^[^ ^11^ ^]^ In the proliferative phase, excessive exogenous mechanical load activates the mechanical force response signaling pathway represented by Yes‐associated protein (YAP). In particular, the fibroblast population is sensitive to mechanical stimulation, such that En1 lineage‐negative fibroblasts (ENF) are transformed into En1 lineage‐positive fibroblasts (EPF), thus promoting high expression of fibrosis‐related genes.^[^ ^7^ ^]^ Meanwhile, EPF cells also induce activation of cells such as keratinocytes and endothelial cells through direct cell‐to‐cell contact and/or indirect activities like paracrine secretion, which secretes and produces more vascular endothelial growth factor (VEGF),^[^ ^12^ ^]^ thus regulating the continuous and high rate of increase in vascularity in the wound. This will mislead the positive and negative feedback coordination mechanisms of the organism, resulting in excessive proliferation of fibroblasts and over‐vascular reconstruction at the wounds. Furthermore, receptors on the surface of fibroblasts recognize excessive exogenous mechanical stimuli which drive fibroblasts to excessively shift to activated phenotypes, namely massive differentiation into highly contractile α‐SMA‐positive myofibroblasts.^[^ ^13^ ^]^ This will overdrive collagen hardening and mediate fibrotic repair of wounds. The deposition of substantial amounts of fibrotic tissue results in the deformation of the epidermal and dermal structures of the nascent skin, leading to the formation of hyperplastic scarring.^[^ ^14^ ^]^

In the face of the dilemma of sports wounds prone to marginal stress concentrations, it is possible to intervene and modulate exogenous mechanical stimuli in independent or synergistic ways by the strategy of blocking mechanical signals transmission within the tissue or unloading external tension.^[^ ^15^ ^]^ In this regard, drugs or gene knockouts are available to block the transduction of mechanical signals into chemical molecule signals. For instance, the use of verteporfin to intervene and inhibit the emergence of YAP results in flaccid paralysis in wound cells and inhibition of mechanical signal transduction.^[^ ^16^ ^]^ This invasive intervention treatment plan carries painful discomfort and risk of postoperative complications. Conversely, the implementation of intelligent physical intervention strategies to dynamically modulate the margin stresses of wounds is a more favorable alternative.^[^ ^17^ ^]^ In view of this, we observed and conducted in‐depth analysis of the energy conversion mechanisms of frogs and kangaroos during jumping, while also considering the need for flexibility of dressings for joint wounds at movement sites. We designed a dual‐network smart hydrogel with both flexible and rigid topological networks. Among them, the rigid topological network disperses and homogenizes the stress at the edge of sports wounds onto the topological network polymer chains of the hydrogel through elastic deformation, that is, transferring energy and storing it in the hydrogel in the form of elastic potential energy. Additionally, an appropriate amount of flexible topological network can endow the hydrogel with certain toughness, inhibit the occurrence and propagation of microcracks under frequent strain, ensure that the hydrogel can alleviate the stress at the wound edge for a long time, and reduce the discomfort caused by dressing replacement. This can also ensure appropriate energy dissipation, inhibit the stress relaxation and creep behavior of the hydrogel, endow the hydrogel with high‐stability elastic potential energy storage capacity, so as to mediate the hydrogel to exhibit stable and continuous high elastic recoil ability.

In this study, inspired by the energy conversion behavior of biological tendon fibers, flexible dynamic chemical bonds and rigid covalent bonds were used to construct the elastic potential energy‐driven shrinkage hydrogel for managing the healing of chronic sports wounds. It can rapidly eliminate drug‐resistant bacteria and their biofilms, alleviate the inflammatory cascade response of the immune system triggered by the bacteria, and coordinate the polarization of macrophages, thereby shortening the stage of inflammatory response of the wound and promoting the wound to enter the proliferation and repair phase. Moreover, the bacterial capture capacity and long‐term antimicrobial ability of the hydrogel anchors periwound bacteria to the surface of the hydrogel and kills them, thereby establishing continuous infection defense lines for the wound. Concurrently, the elastic deformation of the polymer chains within the microscopic rigid topological network is utilized to homogeneously disperse the stresses at the edge of sports wounds and store them in the form of elastic potential energy within topological networks, which in turn dynamically programs the mechanical stimulation transduction of wounds. Overall, this study develops multifunctional programmable elastic recoil hydrogels with bacterial capture properties, long‐term antibacterial properties, repeated antibacterial properties, anti‐biofilm properties, microfluidity properties, self‐healing properties, and on‐demand removal properties, which provide constructive therapeutic solutions for drug‐resistant bacterial‐infected sports wounds.

## 2 Results and Discussion

### 2.1 Design Concept of Mechanically Active Shrinkage Hydrogel with Self‐Adaptive Properties

**Figure 1:**
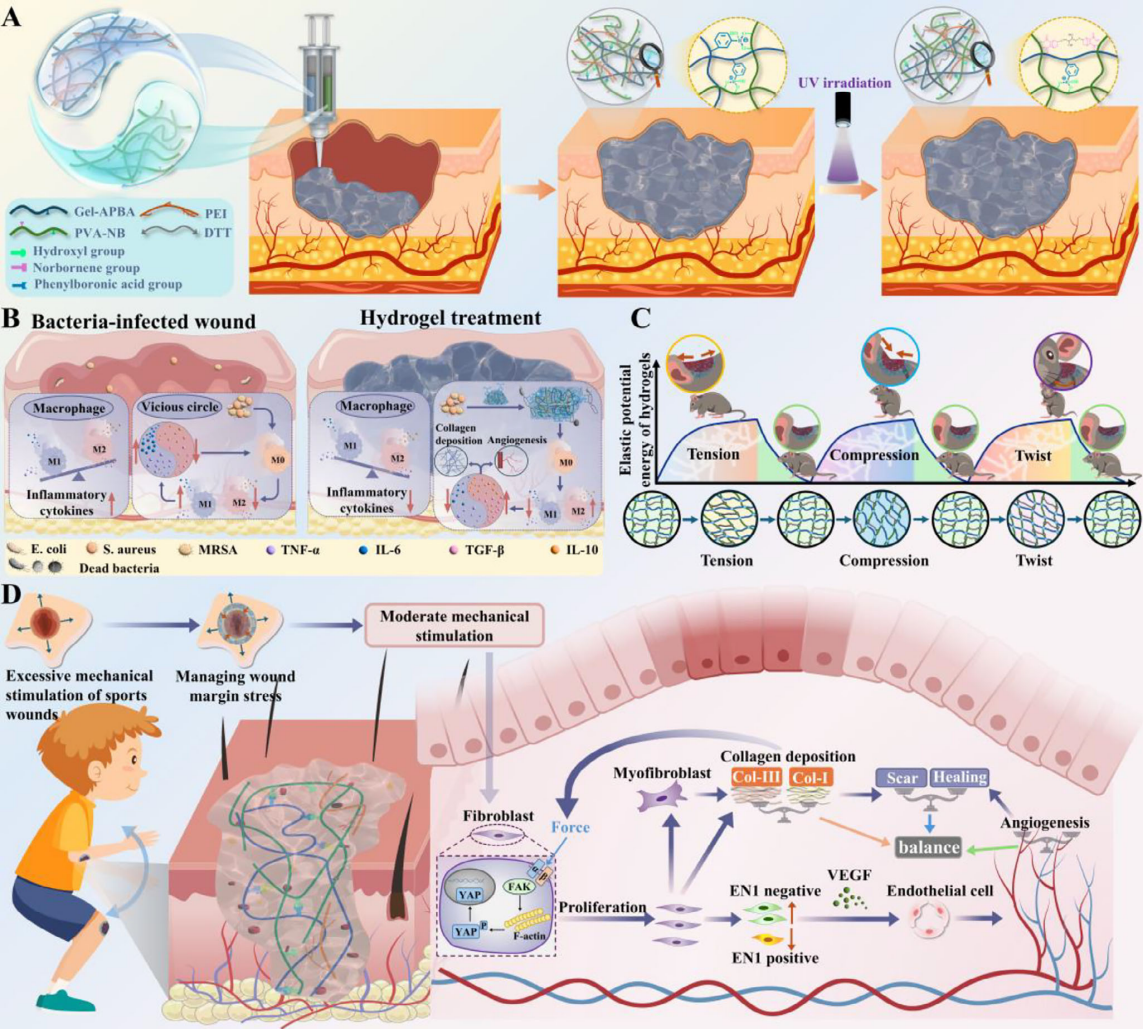
A) Application procedures for hydrogels, B–D) Mechanisms for managing inflammation and mechanical irritation in infected sports wounds.

During the jumping process of frogs and kangaroos, their tendons can achieve efficient energy regulation through unique mechanical behaviors. The core of this mechanism lies in utilizing the elastic properties of tendons to accomplish the mutual conversion between mechanical energy and elastic potential energy. In particular, during the continuous jumping process of kangaroos, when they land, their body weight and inertia cause the tendons of their hind limbs to be stretched (similar to springs being compressed upon landing). ln this process, the strong tendons act like super‐strong springs, converting most of the kinetic energy upon landing into elastic potential energy for storage. Subsequently, when kangaroos jump, their tendons quickly rebound like springs, efficiently releasing stored elastic potential energy as kinetic energy to generate powerful forward propulsion. This cycle of “stretching‐energy storage, rebounding‐energy release” ensures low energy loss during energy conversion in the movement process. Meanwhile, the multilevel topological structure of tendons (such as the braided network of collagen fiber bundles) can disperse the concentrated stress generated by muscle contraction to the entire tissue, preventing damage caused by excessive local stress. For example, the cross‐fiber network of kangaroo tendons can uniformly transmit the instantaneous stress during jumping from the joint edge to the entire tendon unit. This can significantly avoid stress concentration and achieve stress gradient dispersion. Based on the observation and in‐depth analysis of this natural mechanism, we have developed the highly reboundable energy‐carrier hydrogel for treating irregular chronic wounds infected with drug‐resistant bacteria at motion sites. The specific design concept is delineated as follows (**Figure** **1**). Elastic solid hydrogel with viscous fluid properties is exploited by the reversible fracture‐reorganization property of dynamic chemical bonds. It exhibits excellent microfluidic properties to intelligently self‐adapt to complex and variable irregular wounds, thereby implementing efficient and seamless management of irregular wounds at the site of motion. UV irradiation was further utilized to confer the rigid topological network of microflowable hydrogel that fits seamlessly to wounds and meets the needs of sports wounds for dressings (Figure 1A). Wherein, the hydrogel not only can quickly break the hydration layer on the tissue surface through micro‐fluidity to seamlessly fit the intricate wounds, laying the foundation for the establishment of extensive interaction forces at the tissue‐hydrogel interface. It can also rapidly capture floating bacteria at the site of the wound (utilizing the ability of phenylboronic acid to bind to diols in the bacterial cell wall) and remove the bacteria and their debris from the wound. The bacterial capture ability combined with the long‐term antibacterial capacity of the hydrogel not only mitigates the bacterial stimulation to the immune inflammatory response of the organism, thus coordinating macrophage polarization and shortening the span of the inflammatory response (Figure 1B). It also builds prolonged infection defenses lines to safeguard the trauma repair procedure. In addition, the cross‐linked matrix constructed by rigid topological networks exhibits tendon‐mimetic energy conversion properties. This not only can homogenously disperse the stress at the wound edges, avoiding secondary damage to the wound caused by excessive local stress, but also can build the “energy transit station” to transfer the energy generated by traction of sports wounds and store it in the form of elastic potential energy within the internal topological network of the hydrogel (Figure 1C). Afterward, the topological network releases energy through its high resilience, providing appropriate bionic contraction for the wounds. This can effectively shield exogenous mechanical stimuli by inhibiting mechanical signal transduction (YAP), which inhibits the conversion of En1 lineage‐negative fibroblasts (ENF) into En1 lineage‐positive fibroblasts (EPF). This can avoid EPF cells from over‐inducing the activation of cells such as keratinocytes and endothelial cells through direct cell‐to‐cell contact and/or indirect activities like paracrine secretion. This process prevents excessive secretion and production of vascular endothelial growth factor (VEGF), thus achieving the purpose of regulating the collagen fiber arrangement, fibroblasts proliferation and differentiation, and vascular remodeling. Meanwhile, this prevents fibroblasts from excessively transforming to the activated phenotype, namely mass differentiation into highly contractile α‐SMA‐positive myofibroblasts, so as to prevent collagen sclerosis and mediated wound fibrotic repair (Figure 1D).

### 2.2 Preparation and Characterization of Hydrogel

The mechanically active shrinkable hydrogel is formed by mixing 6% (w/v) Gel‐APBA and 8% (w/v) PVA‐NB in equal volume ratios to form the weakly cross‐linked hydrogel with microfluidic properties. Subsequent UV cross‐linking facilitates the transformation of hydrogels with viscous fluid properties into elastic solid hydrogels with superior rebound properties. In this study, Gel‐APBA was prepared by amidation reaction. Specifically, Gel‐APBA was prepared by reacting 3‐aminophenylboronic acid with the carboxyl group on gelatin, catalyzed by EDC‐HCl/NHS (Figure S1A, Supporting Information). Initially, it was characterized by ^1^H NMR. As illustrated in Figure S1B (Supporting Information), the Gel‐APBA spectrum exhibited characteristic peaks (7.40–7.80 ppm) corresponding to 3‐aminophenylboronic acid as compared to the gelatin spectrum. Furthermore, Gel‐APBA was qualitatively and quantitatively analyzed using the characteristic UV absorption peak of the *π*–π* transition of the benzene ring in 3‐aminophenylboronic acid, and the amount of modification of 3‐aminophenylboronic acid was found to be 7.59 wt.%. Moreover, norbornene anhydride is modified onto the main chain of PVA by esterification reaction (Figure S2A, Supporting Information). The modification of norbornene anhydride not only confers the PVA main chain double bonds for further cross‐linking. Concurrently, the opening of the anhydride in the norbornene anhydride provides extensive carboxyl groups to the PVA main chain, thereby enhancing the ability of the PVA main chain to bind water molecules and thus strengthening the water retention properties of the hydrogel it constitutes. Upon comparison of the ^1^HNMR spectrums (Figure S2B, Supporting Information), it is evident that the PVA‐NB spectrum exhibits new characteristic peaks attributed to ‐CH═CH‐ (6.2 ppm), ‐CH‐ (3.3 ppm), ‐CH‐ (3.1 ppm), ‐CH_2_‐ (1.3 ppm), as compared to the ^1^HNMR spectrum of PVA.^[^ ^18^ ^]^ The amount of grafting of norbornene anhydride was quantified by comparing the integral values of particular signal peaks quantitatively, and the degree of substitution was analyzed to be 21.5 wt.%. Since the PVA selected is type 1799, it is the linear macromolecule with 1700 repeating units. The calculation of the degree of substitution indicates that the PVA‐NB utilized in this study provides ≈361 cross‐linking reaction sites per chain. The above results qualitatively illustrate the successful preparation of Gel‐APBA and PVA‐NB, and also quantitatively indicate the amount of their modifications, respectively.

### 2.3 Evaluation of Basic Properties of Hydrogel

**Figure 2:**
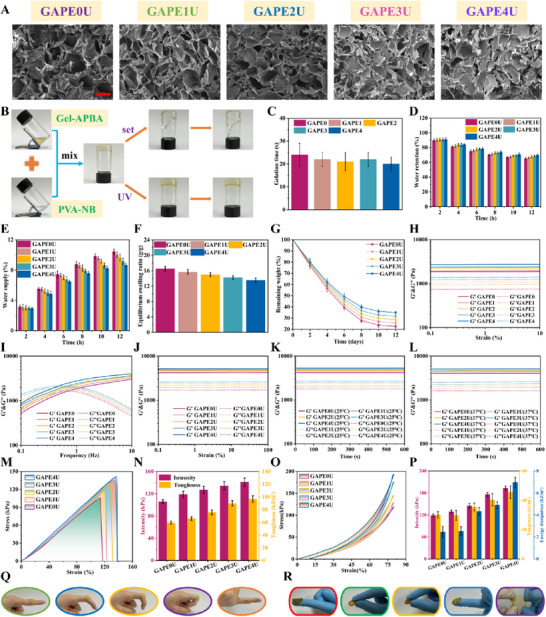
Basic performance of hydrogels. A) Morphological analysis (scale bar: 100 µm), B) Gel‐forming mechanism, C) Gelation time, D) Water retention properties, E) Water supply properties, F) Equilibrium swelling rate, G) Degradation properties, H,J) Strain sweeps, I) Frequency sweeps, K,L) Time sweeps, M,N) Uniaxial stretching with analysis, O,P) Uniaxial compression with analysis, Q,R) Macroscopic tests. Data are mean ± standard deviation (*n* ≥ 3).

The procedure for the preparation of mechanically active shrinkable hydrogels is shown in **Figure** **2**B. When equal volumes of 8% (w/v) PVA‐NB solution and 6% (w/v) Gel‐APBA solution (which respectively contain 0.00%, 0.45%, 0.70%, 0.95%, and 1.20% oligo‐polyethyleneimine) are mixed, it can be found that they can form the hydrogel through dynamic borate ester bonds, which are respectively named GAPE0, GAPE1, GAPE2, GAPE3, and GAPE4. Notably, the weakly cross‐linked hydrogel based on dynamic borate ester bonds exhibits excellent microfluidic properties. The intelligent shape‐adaptive capability of the hydrogel enables it to conform to the intricate shapes of wounds. Subsequent to undergoing UV cross‐linking, the viscous fluid hydrogel was transformed into the elastic solid hydrogel, which are respectively named GAPE0U, GAPE1U, GAPE2U, GAPE3U, and GAPE4U. Their excellent flexibility provided strong guarantee for sports wounds. Additionally, the microscopic 3D network of the GAPEU hydrogels was observed by scanning electron microscopy. As observed (Figure 2A), the hydrogels exhibited the uniform and through‐pore structure, while the average pore size of the hydrogels gradually decreased with the increase of oligo‐polyethyleneimine content in the system. This phenomenon can be attributed to the increase in cross‐linking density of the topological network of the system, resulting in the denser internal pore structure. The hydrogel demonstrated a superior in situ gel‐forming ability, namely the short gelation time, which is essential for the convenient clinical application of the hydrogel. As illustrated in Figure 2C, a series of weakly cross‐linked hydrogels have relatively short gelation times. This observation signifies not only the simplicity of application of the hydrogels but also their notable gelation capacity. The wound healing procedure demands the warm and moist external environment to facilitate the migration and proliferation of fibroblasts. It requires dressings to provide and maintain the moistness of the external wound environment, namely, to possess favorable water‐retention and water‐supply capabilities. In this case, the water retention capacity of a series of hydrogels was evaluated in the open environment at room temperature. Remarkably (Figure 2D), the water retention of the hydrogels was still above 65% even after 12 h, providing the solid foundation for the hydrogels to continuously supply water. In addition, the water supply capacity of a series of hydrogels was tested with simulated skin. As illustrated in Figure 2E, the GAPE3U and GAPE4U hydrogels provided more than 8% water to the simulated skin after 12 h. The above results provide strong evidence for the ability of the hydrogels to provide sustained moisturization of the external wound environment. Tissue exudate is an unavoidable occurrence in cases of open damaged tissue during the process of wound healing. This requires dressings with immediate exudate removal and prolonged wound exudate management, namely excellent swelling capacity. As shown in Figure S3 (Supporting Information), a series of hydrogels exhibited rapid water absorption rates in the initial phase, suggesting that the hydrogels possessed the ability to remove exudate quickly and immediately. As expected, the hydrogels attained equilibrium swelling levels after 20 h (Figure 2F), with equilibrium swelling rates of 16.48 g/g (GAPE0U), 15.68 g/g (GAPE1U), 14.97 g/g (GAPE2U), 14.23 g/g (GAPE3U), and 13.53 g/g (GAPE4U), respectively. Subsequent analysis revealed that the equilibrium swelling rate of a series of hydrogels decreased as the solid content of oligo‐polyethyleneimine in the system increased. This phenomenon can be attributed to the ability of the oligo‐polyethyleneimine to form extensive hydrogen bonds with the Gel‐APBA and PVA‐NB molecular chains in the system, thereby increasing the density of the hydrogel topological network. This results in an increase in the cohesive energy of the cross‐linked network, which macroscopically exhibits slightly lower equilibrium swelling rates. Furthermore, it is imperative that dressings providing the temporary protective barrier for open tissues have degradation rates consistent with tissue regeneration. To this end, the degradation performance of a series of hydrogels was evaluated in simulated in vivo physiological environmental systems (PBS, pH = 7.4). The residual weights of a series of hydrogels during the trauma remodeling period (Figure 2G) were 22.56% (GAPE0U), 25.75% (GAPE1U), 28.63% (GAPE2U), 31.52% (GAPE3U), and 34.89% (GAPE4U). The aforementioned data thoroughly demonstrate that hydrogels have degradation rates consistent with the regenerative remodeling of traumatic tissues.

### 2.4 Viscoelastic Properties and Mechanical Performance of Hydrogel

Skin is a complex and mysterious “hydrogel material”. It exhibits the microfluidic properties of the viscous fluid and the flexibility of the elastic solid. Consequently, the ideal dressing for transient use as the skin substitute is the bionic material with both viscous and elastic properties. Initially, the topological network condition of the hydrogels was evaluated by rotational rheometry, namely testing the energy storage modulus (G′) and loss modulus (G″) of the hydrogels. As demonstrated in Figure 2H, at the constant frequency of 1 Hz, the energy storage modulus invariably exceeds the loss modulus (G′ > G″) during the dynamic strain scan (0.1%–10%) of the weakly cross‐linked hydrogel based on dynamic borate ester bonds. This finding suggests that the molecular chains within the hydrogel system have undergone cross‐linking, resulting in the formation of a 3D network structure. Compared to this, following UV cross‐linking, a series of hydrogels exhibited higher energy storage modulus in the dynamic strain scans (Figure 2J), indicating that the hydrogels possess superior flexibility. Concurrently, the GAPEU hydrogel manifests broader linear viscoelastic zones, indicating its capacity to withstand larger strains without being destroyed. In addition, the stability of the GAPEU hydrogel was examined at a constant temperature of 25 °C. It is evident that G' is consistently greater than G″ during 600 s (Figure 2K), signifying that the topological network of the hydrogel remains intact. The constant physiological temperature (37 °C) condition was selected for time scanning of the GAPEU hydrogel. Notably (Figure 2L), the energy storage modulus and loss modulus of the hydrogel did not exhibit substantial fluctuations within 600 s, thereby strongly suggesting the stability of the topological network structure within the hydrogel. The above results demonstrate that the GAPEU hydrogel possesses a wide linear viscoelastic zone and excellent stability of mechanical properties, which can meet the demand for flexible dressings for sports wounds. To further illustrate the hydrogels possessing excellent mechanical properties. Initially, the mechanical properties of the hydrogels were evaluated using the uniaxial tensile program (Figure 2M). As demonstrated in Figure 2N, the breaking strength of the GAPEU hydrogel increases from 105.91 to 141.05 kPa with increasing solid content of oligo‐polyethyleneimine, as well as the toughness from 59.41 to 96.71 kJ m^−3^. The phenomenon may be attributed to oligo‐polyethyleneimine, which is uniformly dispersed in the system, can construct the second‐layer network of the hydrogel with the molecular chains of PVA‐NB and Gel‐APBA through the interaction forces such as hydrogen bonding, resulting in denser topological network matrices, and thus exhibiting superior mechanical properties. Furthermore, the construction of the second‐layer network facilitates the rapid transfer of stresses, thereby enabling the network to withstand stronger applied loads. On the other hand, the second network in the system is mainly constructed by interaction forces such as hydrogen bonding that can rapidly dissipate and rebuild, thus alleviating the defects of stress concentration, dispersing the energy, and thus improving the toughness of GAPEU hydrogels. Subsequently, uniaxial compression tests at 80% were also applied to the GAPEU hydrogel. The compression curve rises smoothly, as illustrated in Figure 2O, indicating that the hydrogel did not undergo plastic damage. The findings demonstrate the hydrogel's capacity to withstand substantial compressive strains without permanent damage. At an 80% strain, the compressive stress of the GAPEU hydrogels exhibited results consistent with the breaking stress. Interestingly, the cyclic compression curves exhibited larger hysteresis loops with increasing oligo‐polyethyleneimine additions, as revealed by the analysis. The increase in hysteresis loop of GAPEU hydrogel indicates its enhanced energy dissipation capability. It is attributed to the second network constructed by the oligo‐polyethyleneimine with PVA‐NB and Gel‐APBA molecular chains through interaction forces such as hydrogen bonding, which transforms from the entangled to the disentangled state. The procedure consumes partial energy, thus other GAPEU hydrogels exhibit more significant hysteresis loops compared to GAPE0U hydrogel, but it can also provide additional toughness to the GAPEU hydrogel. The above results demonstrate that GAPEU hydrogel possesses favorable flexibility, which can meet the motion needs of joint skin.

Currently, the majority of hydrogels applied to skin repair for joint injuries exhibit elastic solid properties, presenting stable and flexible topological network structures. However, skin injuries tend to exhibit irregularities, requiring the hydrogel also possess the properties of viscous fluid, capable of micro‐flow to adapt to irregular wounds. As can be seen in Figure 2I, during low‐frequency sweeps, G″ > G′. This indicates that the relaxation time (τ) of the cross‐linking points in the internal 3D network of the hydrogel is shorter than the duration of external force action. At this point, the cross‐linking points of the topological network frequently break and reform, which causes the hydrogel to exhibit microfluidic properties. In contrast, during high‐frequency sweeps, G′ > G″. This indicates that the relaxation time (τ) of the cross‐linking points in the hydrogel network is longer than the duration of external force action. At this point, the internal cross‐linking points of the topological network behave as fixed nodes, causing the polymer chains in the system to be stretched like “springs” without slipping, thus making the hydrogel exhibit solid‐like elasticity.

In this context, the practicality of applying GAPEU hydrogel to joint skin was assessed. GAPE hydrogel was injected at the experimenter's knuckle and further cross‐linked in situ to form GAPEU hydrogel (Figure 2Q). GAPEU hydrogel can firmly accompany the finger to move freely without damage or falling off, visually verifying that the hydrogel has excellent flexibility and adhesion properties. Moreover, by shaping the GAPEU hydrogel into a disk‐like form and then folding it, the hydrogel did not break and exhibited rapid recovery (Figure 2R). This finding indicates that the GAPEU hydrogel can withstand high levels of bending. Subsequent bi‐directional stretching of the disc‐shaped GAPEU hydrogel vividly and intuitively revealed that the hydrogel has favorable mechanical properties.

### 2.5 Fatigue Resistance and Elastic Potential Energy Storage Capacity of Hydrogel

**Figure 3:**
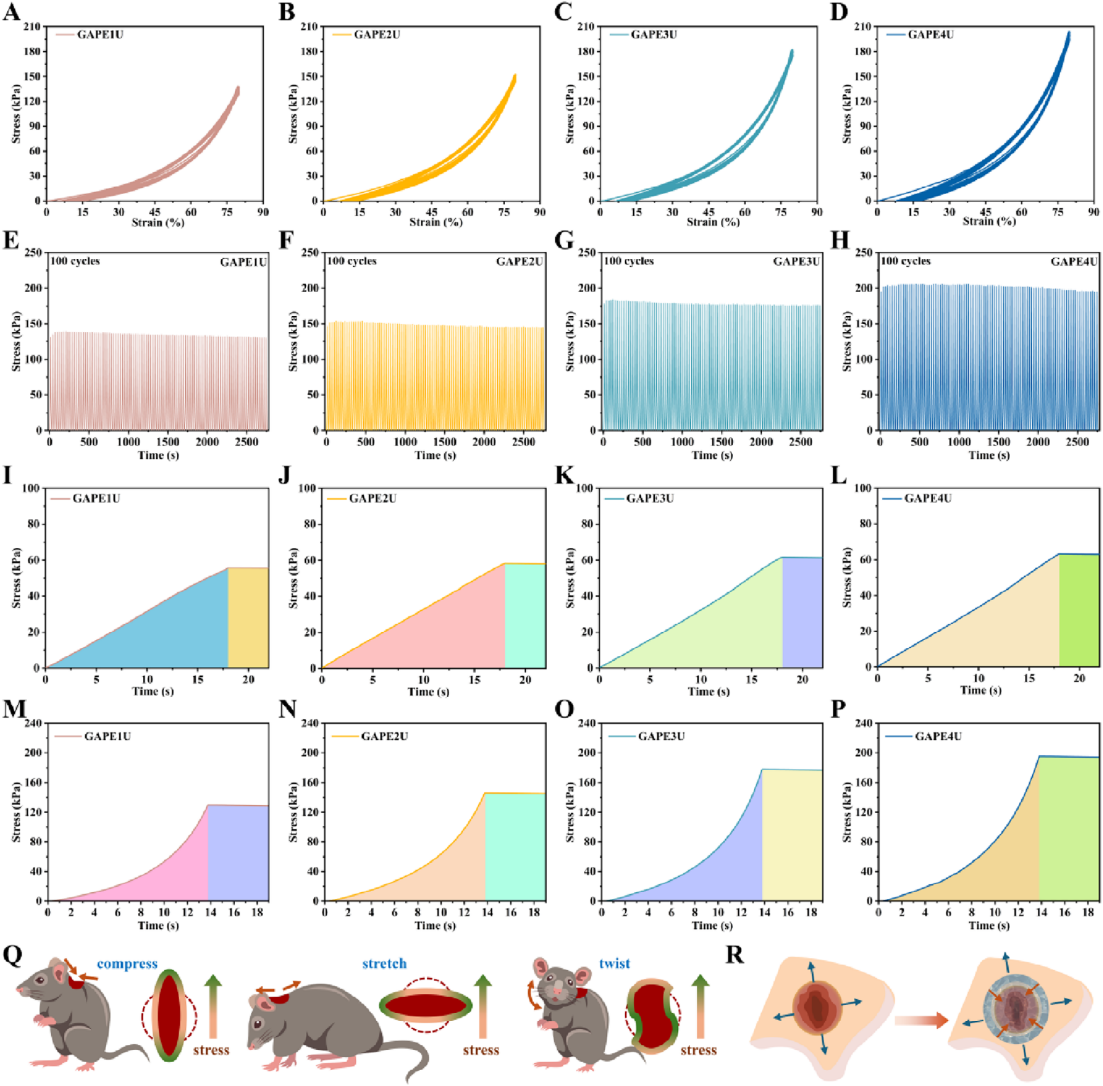
Fatigue resistance and elastic potential energy storage ability of hydrogels. A–D) Hundred compression cycles, E–H) Time‐stress analysis, I–L) Static tension, M–P) Static compression, Q) Stress distribution at the edge of the athletic wound, and R) Management by hydrogel on the athletic wound. Data are mean ± standard deviation (*n* ≥ 3).

Sports wounds are subject to frequent pulling, bending, and twisting. This requires dressings to possess not only favorable mechanical properties, but also the ability to maintain excellent mechanical properties, namely superior fatigue resistance. The fatigue resistance of GAPEU hydrogels was evaluated with 100 uninterrupted compression loading‐unloading tests. As illustrated in **Figure** **3**A–D and S5A (Supporting Information), the loading‐unloading curves did not exhibit any defects, thereby demonstrating that the topological network of a series of GAPEU hydrogels did not collapse dramatically under repeated compression. Furthermore, the stress and energy dissipation of the second cycle was observed to be reduced. This phenomenon is attributed to the time available for repairing the internal topological network of the hydrogel in the uninterval test being limited, resulting in partial cross‐linked network matrices not being recovered. The subsequent compression cycle curves essentially overlap, showing minimal difference from the initial compression cycle. This aspect suggests that most of the second network consisting of hydrogen bonds within the hydrogel can be quickly reconstructed, namely the network is well reconfigurable. Additionally suggests the hydrogel internal network possesses stable reconfigurability, namely indicates the second network fracture‐reconfiguration has excellent stability and repeatability, namely suggests the hydrogel has the stable and repairable energy dissipation ability. The compressive stress‐time of loading‐unloading in hundred uninterrupted compression was further analyzed (Figure 3E–H; Figure S5B, Supporting Information). Notably, the stress decay after one hundred compressions was negligible, always maintaining more than 95% of the initial stress value. These results strongly suggest that GAPEU hydrogels possess excellent elastic recovery ability and fatigue resistance properties. Significantly, the GAPEU hydrogel exhibits comparatively narrow hysteresis lines, suggesting that the hydrogel dissipates less energy during compressive loading‐unloading processes, which indicates that the majority of the topological network of the hydrogel is composed of stable covalent bonds. The rigid topological network would confer the hydrogel with favorable elastic recoil capacity, namely excellent resilience properties. For further validation, a series of GAPEU hydrogels were subjected to static compression (Figure 3M–P; Figure S5C, Supporting Information) and static tensile testing (Figure 3I–L; Figure S5D, Supporting Information). After applying the specific strain, the hydrogel displayed no significant decrease in stress during the static test, suggesting that the hydrogel possesses notable elasticity. This also indicates the network constructed by covalent bond cross‐linking has excellent rigidity, which inhibits the stress relaxation behavior of the network and endows the hydrogel to exhibit excellent elastic recoil capacity. Repeated pulling of sports wounds (Figure 3Q) results in greater stress on the wound edges, leading to recurrent bleeding and massive exudates in the initial wound, which are particularly prone to the growth of microorganisms such as bacteria, thus disrupting the normal wound healing procedure. In addition, for late‐stage wounds, the stress concentration resulting from repeated pulling can overstimulate the wound healing cascade response, resulting in excessive cell multiplication, hyper‐angiogenesis, and collagen proliferation, thus causing the formation of scars. When GAPEU hydrogel is applied to sports wounds (Figure 3R), the excellent rebound properties confer mechanically adaptive rebound force to the hydrogel, which dynamically programs the wound edge stresses, thus alleviating the abnormal healing procedures due to exercise and strongly preventing scarring.

### 2.6 Injectable, Self‐Adaptive, and Self‐Healing Properties of Hydrogel

The uncertainty of the danger results in wounds occurring at the joints of the body, and most of them present longitudinal, narrow, and deep wounds, which require dressings with favorable injectability.^[^ ^19^ ^]^ The viscosity of the GAPE hydrogel was shown to decrease drastically with increasing shear rate by rotational rheometer tests in this study, indicating excellent shear‐thinning behavior (Figure S7A, Supporting Information). Furthermore, the GAPE hydrogel was injected into the syringe, and the letter “HL” was written down, indicating visually the excellent injectability of the hydrogel.

In the case of wounds that are longitudinally narrow and deep, it is necessary for hydrogels to possess both injectable properties and the capacity to adapt to the wounds' intricate shapes.^[^ ^20^ ^]^ In this experiment, the microfluidity properties of the hydrogel were assessed by placing the GAPE hydrogel in the small beaker containing glass beads. As illustrated in Figure S8 (Supporting Information), the hydrogel can adaptively fill four layers of glass beads and their gaps within 66 min under the effect of gravity. This phenomenon intuitively demonstrates that GAPE hydrogel possesses favorable viscous fluid properties and can intelligently adapt to wounds with intricate gaps, thus providing seamless and efficient wound care. Furthermore, when it comes to athletic injuries, hydrogels must not only adapt to the diverse shapes of wounds but also possess superior elastic properties, which can program wound edge stresses dynamically and coordinate the regulation of the wound healing cascade from mechanical stimuli. To this end, the elastic properties of GAPEU hydrogels, constructed with rigid covalent bonded cross‐linked networks, were further assessed through simulation experiments. The GAPE hydrogel, which is microflowing, is endowed with a rigid cross‐linking structure of the internal topological network through UV intervention. Subsequently, the hydrogel no longer microflows to intelligently adapt to intricate pores, indicating the transformation of the hydrogel from visco fluid‐like to pliable elastic solid. The above results demonstrate that GAPE hydrogel exhibits remarkable viscous fluid properties, while GAPEU hydrogel displays exceptional elastic solid properties. A series of GAPEU hydrogels can intelligently and adaptively cover wounds, thereby providing a skin‐like barrier and efficiently orchestrating the healing process. Additionally, these hydrogels can be constructed to possess rigid topological network structures, thus intelligently and dynamically programming sports wound edge stresses.

In order to comprehensively evaluate the injectable and self‐adaptive properties of hydrogels. Here (Figure S7B, Supporting Information), long irregular deep wounds were fabricated on the surface of porcine liver, which was used to evaluate the convenience of hydrogel application. Evidently, the hydrogel can be easily injected into the long, irregular, deep wound through a syringe. After 15 min of standing, the hydrogel has intelligently adapted to the complicated irregular wound. After further endowing the internal topological network with a rigid cross‐linking structure. After washing with water and bending the porcine liver, the hydrogel was found without fragmentation and shedding. The above results demonstrated that the hydrogel exhibited favorable injectability, microfluidity, flexibility, and adhesion properties, thereby substantiating the significant potential of GAPEU hydrogel for clinical application.

Due to frequent stretching and bending, it is inevitable that dressings applied to joints will crack. Therefore, dressings must possess suitable self‐healing capabilities. As shown in Figure S6G (Supporting Information), the self‐healing ability of the hydrogel was first macroscopically evaluated. Semi‐circular hydrogels containing different pigments were placed tightly together and left to stand for a period of time. After suitable stretching, no separation was observed. This phenomenon intuitively suggests that part of the topological network has been reconstructed. To quantify the reconstruction of the topological network, namely the self‐healing rate, the self‐healed samples were subjected to uniaxial tensile tests (Figure S6A–E, Supporting Information). The self‐healing rate of the hydrogel was quantitatively revealed to be 52.96% (GAPE0U), 53.21% (GAPE1U), 53.79% (GAPE2U), 54.45% (GAPE3U), and 55.11% (GAPE4U), respectively. Hydrogels exhibit limited self‐healing ability because of the unreconstructability of covalent bonds. The self‐healing mechanism is illustrated in Figure S6H (Supporting Information). This process is primarily attributed to the reconstruction of dynamic borate ester bonds at the interface, while interaction forces, such as hydrogen bonding between polar groups at the interface, also contribute to the reconstruction of the 3D network.

### 2.7 Adhesion Properties of Hydrogel

**Figure 4:**
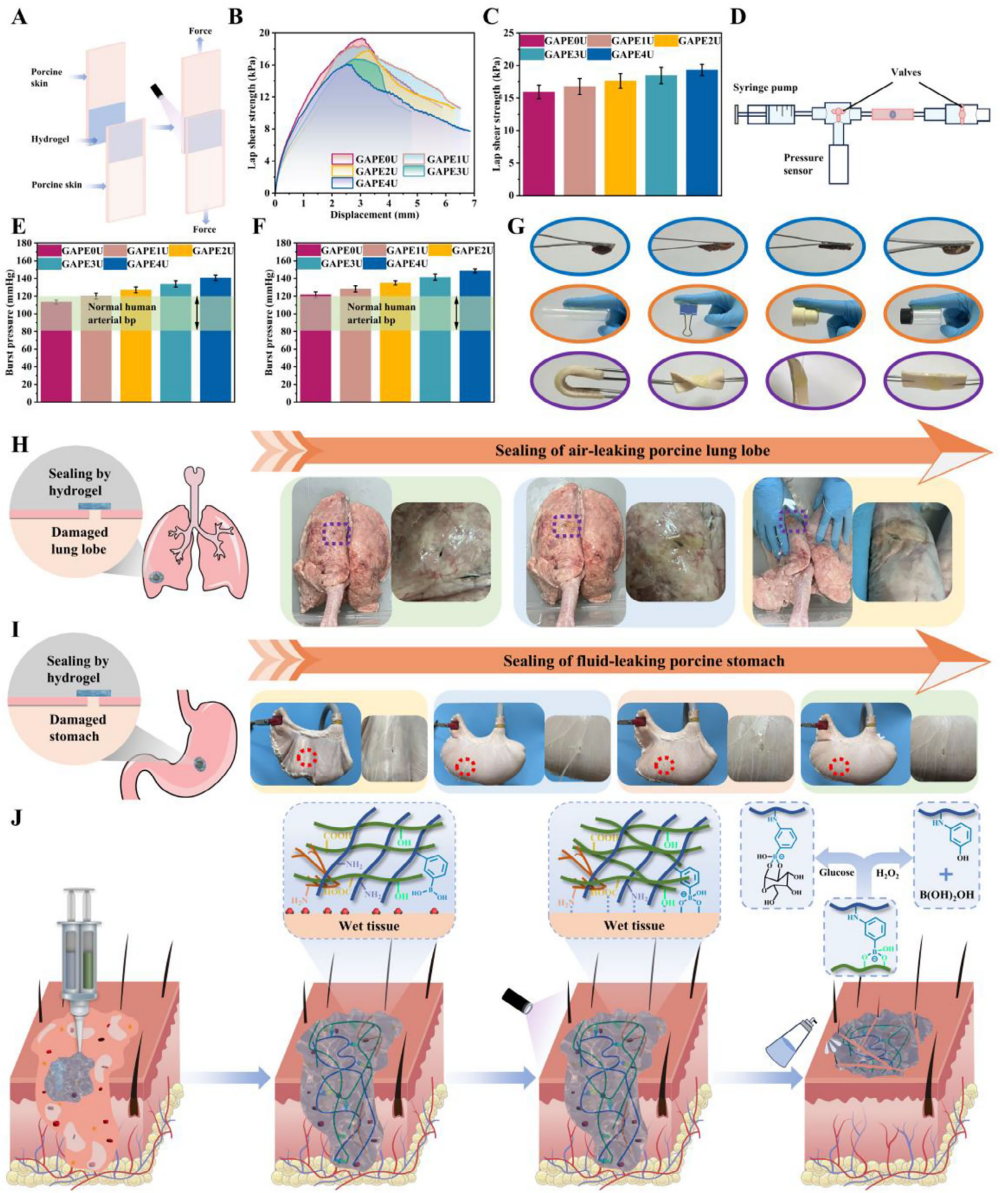
Adhesion properties of hydrogels. A–C) Schematic and statistical graphs of lap shear. D–F) Schematic and statistical graphs of bursting test, G–I) Adhesion physical diagrams, J) Diagram of adhesion‐detachment mechanism. Data are mean ± standard deviation (*n* ≥ 3).

Injuries in the joint area undergo healing procedures with the much higher frequency of movement than static wounds. This requires the dressing to have exceptional adherence properties to ensure that it remains firmly attached to the wound and continues to dynamically coordinate the wound healing process. Initially, the adhesion properties of the GAPEU hydrogel were tested macroscopically. As shown in **Figure** **4**G, the skin with hydrogel adhesion was violently twisted, finding the hydrogel did not detach or rupture. Even after rinsing with water, the GAPEU hydrogel remained firmly attached to the skin surface. The performance of the hydrogel in terms of adhesion to wet organs was further evaluated. Apparently, the GAPEU hydrogel was able to adhere and lift wet heart, liver, spleen and kidney. Additionally, the hydrogel could adhere to glass vials (11.35 g), plastic tubes (3.89 g), iron clips (5.01 g), and rubber stoppers (6.72 g) without falling off. And GAPEU hydrogel can effectively seal broken porcine stomachs and leaky porcine lungs (Figure 4H,I). These findings collectively demonstrate the GAPEU hydrogel's broad‐spectrum and exceptional adhesion properties. The adhesion ability of the GAPEU hydrogel was further quantified. First, the skin adhesion properties of the hydrogel were evaluated by lap shear test (Figure 4B,C). The maximum stress at the time of separation between the two pieces of porcine skin with complete adhesion was the adhesion capacity of the hydrogel. The lap shear strengths of a series of hydrogels were 15.93 kPa (GAPE0U), 16.77 kPa (GAPE1U), 17.62 kPa (GAPE2U), 18.45 kPa (GAPE3U), and 19.32 kPa (GAPE4U). Furthermore, the ability of the GAPEU hydrogel to seal wet skin and wet small intestinal injury holes was evaluated using the homemade real‐time pressure monitoring system (Figure 4E,F). A series of hydrogels were found to have a sealing capacity of 121.58 mmHg (GAPE0U), 128.19 mmHg (GAPE1U), 134.94 mmHg (GAPE2U), 141.39 mmHg (GAPE3U) and 148.51 mmHg (GAPE4U) for skin lesion holes that were higher than the arterial blood pressure of the normal human (80–120 mmHg). A series of GAPEU hydrogels exhibited the similar regularity for small intestinal injury holes. Notably, the GAPEU hydrogels displayed high adhesion properties. This can be attributed, on the one hand, to the viscous fluid properties of GAPEU hydrogel that seamlessly fills irregular wounds, thereby allowing extensive surface polar groups to form a mechanically interlocking structure with the tissue. On the other hand, the rigid structure of the internal topological network of GAPEU hydrogel provides excellent cohesion to the hydrogel. Overall, it is the result of the combined effect of cohesive and interfacial interaction forces in the hydrogel topological network. Analyzing the above data, GAPEU hydrogels exhibited superior adhesion properties as the addition of oligo‐polyethyleneimine increased in the hydrogel system. The observed phenomenon can be attributed to the presence of oligo‐polyethyleneimine, containing abundant polar groups, which will help the hydrogel to break the hydration layer on the surface of the wet skin, promoting the formation of a more extensive hydrogel‐skin interaction force. Concurrently, the second network, constructed by its hydrogen bonding interaction force, will increase the rigidity of the network matrix, prompting the hydrogel to exhibit superior cohesive energy.

### 2.8 Performance of Painless Removal of Hydrogel

While it is important for dressings to have excellent adherence, clinical use inevitably involves dressing misplacement and the need for the patient or clinician to regularly monitor the development of chronic wounds. This requires dressings that can be removed without compromising nascent tissue. Here, the removal performance of GAPEU hydrogel was evaluated by testing the removal agent‐treated adherent completed porcine skin and the removal agent‐treated hydrogel‐sealed injury holes by burst tests and lap‐shear tests. Comparison of Figures S10A,B (Supporting Information) revealed that the H_2_O_2_ solution treatment has superior desorption performance. In addition, the evaluation of the blast test showed the similar regularity. Remarkably, the co‐treatment with glucose and H_2_O_2_ solutions used for the porcine skin adhesive interface exhibited a more perfect detachment performance (Figure S10C, Supporting Information), with residual adhesive stresses of 3.99, 4.23, 4.61, 4.76, and 5.08 kPa, and corresponding detachment rates of 74.95%, 74.78%, 73.89%, 74.22%, and 73.71%, respectively. Meanwhile, it was found that GAPEU hydrogel‐sealed porcine skin injury holes co‐treatment by glucose and H_2_O_2_ solutions also exhibited excellent detachment ability (Figure S11C, Supporting Information), with detachment rates as high as 73.59%, 73.14%, 72.11%, 72.29%, and 71.76%, respectively. In addition, glucose/H_2_O_2_ solution also efficiently removed the GAPEU hydrogel sealed on the injury holes of the small intestine (Figure S12C, Supporting Information). The above data suggest that the adhesion ability of GAPEU hydrogel to wet tissue can be efficiently reduced when glucose/H_2_O_2_ solution is used as a removing agent. This can be attributed to the ability of glucose and H_2_O_2_ to disrupt the topological network cross‐linking points, as the mechanism shown in Figure 4J. When the bonding interface between hydrogel and tissue is treated with glucose/H_2_O_2_ solution, small molecules of glucose and H_2_O_2_ rapidly penetrate into the 3D network of the hydrogel. In this case, small molecules of glucose will compete with the polyols in the polyvinyl alcohol backbone to capture and bind the limited number of phenylboronic acid groups, thus disrupting the dynamic equilibrium of the borate ester bonds breakage‐formation process. While H_2_O_2_ directly damages the phenylboronic acid groups and prevents them from reconstructing the topological network cross‐linking points through dynamic chemical bonds. It will lead to the collapse of some topological network cross‐linking points in the hydrogel system, resulting in a decrease in the overall cross‐linking point density of the topological network, thus drastically reducing the cohesion of the hydrogel topological network matrix. This simultaneously causes the extensive interaction forces established at the hydrogel‐tissue interface to fail, thereby down‐regulating the hydrogel‐tissue interfacial interaction forces. The combined decrease in interfacial interaction forces and cohesion results in the weakening of the adhesive capacity of the GAPEU hydrogel, allowing it to be easily removed from the tissue without damaging the nascent granulation tissue. Remarkably, the GAPEU hydrogel removal procedure is relatively simple and the removal agent is biocompatible, providing an excellent basis for convenient clinical application.

### 2.9 Evaluation of Bacterial Capture Performance of Hydrogel

**Figure 5:**
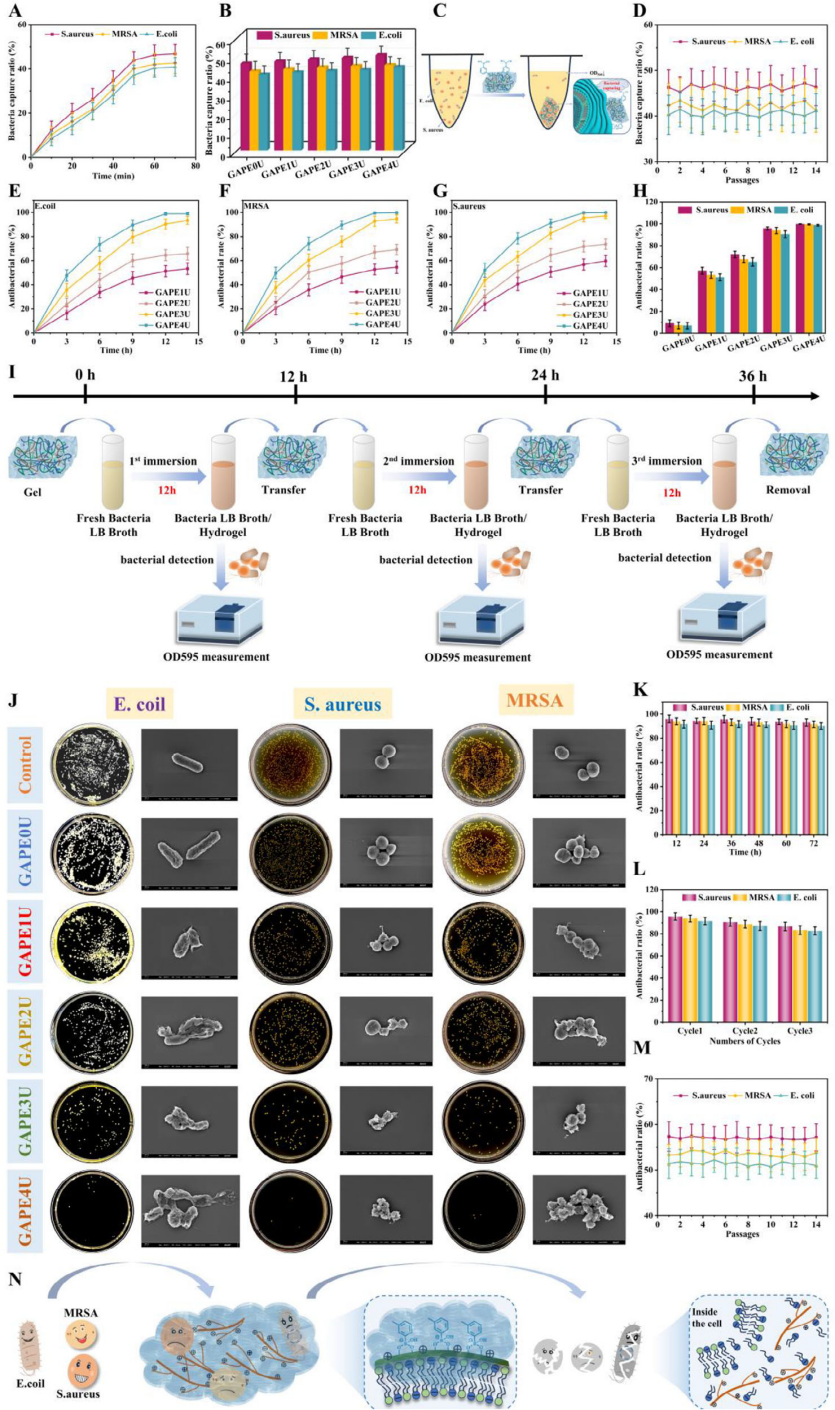
Bacterial capture performance and long‐term bactericidal properties of hydrogels. A–C) Schematic representation of bacterial capture, kinetics, and statistical plots, D) Test of resistance to capture, E–G) Bactericidal kinetics, J,H) Bacterial electron micrographs (scale bar: 200 nm), bacterial agar plates, and statistical plots, I,L) Repeated antibacterial properties, K) Long‐term antibacterial properties, M) Resistance monitoring, N) Antibacterial mechanism. Data are mean ± standard deviation (*n* ≥ 3).

Chronic wounds caused by bacterial infections not only harbor massive amounts of bacteria, but also have large amounts of active bacteria around the wound due to the constant movement and multiplication of bacteria. This requires antimicrobial dressings that not only effectively cleanse pathogenic bacteria from the internal environment of the wound, but also dressings capable of capturing and clearing planktonic bacteria from the periwound area to provide a favorable external environment for wound repair procedures. In this regard, the ability of the GAPE0U hydrogel to capture bacteria was evaluated by selecting as representative strains the causative organisms commonly found in chronic wounds (*E. coli, S. aureus*, and *MRSA*). The capture process of the hydrogel for bacteria was first presented quantitatively by monitoring the optical density values of the supernatant after co‐contact of the hydrogel with the bacterial dilution (**Figure** **5**A). Not surprisingly, the capture rate of the hydrogel for bacteria increased with increasing contact time, but after 60 min, the capture rate no longer increased. This indicates that the hydrogel has reached the equilibrium capture point for bacteria with capture rates of 40.37% (*E. coli*), 46.28% (*S. aureus*), and 42.08% (*MRSA*), respectively. In addition, the capture ability of a series of hydrogels was quantified and compared (Figure 5B). It can be observed that the hydrogels have superior capture performance for gram‐positive bacteria. This result can be attributed to the more complex cell wall structure of gram‐negative bacteria compared to gram‐positive bacteria, which can better resist the adhesion of the hydrogel. Meanwhile, the capture efficiency of the hydrogel for bacteria increased with the increase of oligo‐polyethyleneimine content in the system. It may be that the increased number of polar groups in the hydrogel system provides the hydrogel with more opportunities to generate interaction forces, such as hydrogen bonding and electrostatic forces, with negatively charged bacteria, thereby enhancing the adsorption of the hydrogel to bacteria. The above results indicate that the GAPEU hydrogel has excellent bacterial capturing ability, and its possible bacterial capturing mechanism is shown in Figure 5C. Lipopolysaccharide (LPS)/peptidoglycan (PGN) in gram‐negative and gram‐positive bacteria has abundant hydroxyl groups, which can form dynamic borate ester bonds with phenylboronic acids on the polymer chains making up the topological network structure of the hydrogel, anchoring the bacteria to the surface of the hydrogel, thus reducing the number of free bacteria in the supernatant. The polar groups in the hydrogel also physically adsorb the bacteria to the surface of the hydrogel through physical interaction forces. Moreover, the evolutionary performance of bacteria for hydrogel capturing was investigated, namely whether bacteria develop capture resistance. Here, the captured final bacterial dilutions were centrifuged to collect the bacteria and reincubated to the tested concentration after washing to re‐test the capture ability of the hydrogel. It is observed that the capture efficiency of GAPEU hydrogel for bacteria does not fluctuate much, indicating that the bacteria do not develop capture resistance to the capture performance of the hydrogel.

### 2.10 Evaluation of the Long‐Term Bactericidal Properties, Repetitive Bactericidal Properties, and Antibiofilm Properties of Hydrogel

Open wounds without immediate treatment are particularly susceptible to bacterial colonization, leading to the formation of chronic wounds infected with bacteria. This requires dressings that can eliminate bacteria from the wound and maintain the clean external environment of the wound. In this study, gram‐negative (*E. coli*) and gram‐positive (*S. aureus*) bacteria were selected to test the antimicrobial properties of GAPEU hydrogel. Initially, the hydrogel was co‐cultured with bacterial suspensions, and the bactericidal procedure of the hydrogel was presented quantitatively by monitoring the optical density values of the supernatants. As demonstrated by the bactericidal kinetic curves (Figure 5E‐G), the bacteria co‐cultured with a series of GAPEU hydrogels gradually decreased with increasing co‐culture time. It is evident that the bacteria in the GAPE3U hydrogel and GAPE4U hydrogel treatment groups have been nearly completely eliminated after 12 hours. Furthermore, the antibacterial capacity of a series of GAPEU hydrogels was assessed quantitatively and deeply by the spread plate method (Figure 5J). The GAPE3U hydrogel and GAPE4U hydrogel treatment groups exhibited fewer bacterial colonies, visually demonstrating the superior antibacterial capacity of the GAPEU hydrogel. Statistical analysis (Figure 5H) revealed that the antibacterial rate of the hydrogel against *E. coli* was 6.98% (GAPE0U), 51.11% (GAPE1U), 65.17% (GAPE2U), 90.49% (GAPE3U), and 98.74% (GAPE4U), and its antibacterial rate against *S. aureus* was 9.13% (GAPE0U), 57.16% (GAPE1U), 72.11% (GAPE2U), 95.81% (GAPE3U), and 99.78% (GAPE4U).

As the global environment undergoes changes, common bacteria evolve into drug‐resistant bacteria. This phenomenon results in the inability of commonly used antibiotics to effectively inhibit bacterial growth, which poses a significant threat to human life and production. Methicillin‐resistant *S. aureus* was selected to evaluate the inhibitory effect of a series of GAPEU hydrogels on drug‐resistant bacteria. Initially, the bactericidal procedures of a series of GAPEU hydrogels were groped by the OD method. Furthermore, the capacity of the hydrogels to eradicate *MRSA* was quantitatively assessed by the spread plate method (Figure 5J). The GAPE3U and GAPE4U hydrogels displayed remarkable capabilities in eradicating drug‐resistant bacteria, with antibacterial rates of 93.87% and 99.48%, respectively. The data presented herein indicate that the hydrogels are capable of not only eradicating common gram‐positive and negative bacteria, but also eliminating drug‐resistant bacteria. The phenomenon can be attributed to the positively charged oligo‐polyethyleneimine being enriched on the negatively charged bacterial cell membrane through electrostatic interactions, altering the permeability of the cell membrane and resulting in the leakage of cytoplasm, thereby leading to the death of the bacteria (Figure 5N). It is also apparent from the above data that GAPEU hydrogels exhibit greater antibacterial activity against gram‐positive bacteria. The finding may be due to gram‐negative bacteria possessing more complex cellular structures than gram‐positive bacteria, resulting in slightly enhanced resistance to oligo‐polyethyleneimine. The effect of GAPEU hydrogel on bacterial cell membranes was further intuitively observed using FESEM. Notably (Figure 5J), the bacteria in the control and GAPE0U hydrogel‐treated groups exhibited intact and smooth cell membranes, along with good bacterial morphology, indicating favorable viability of the bacteria. Conversely, bacteria in the GAPE4U hydrogel‐treated group exhibited signs of cell membrane rupture, cytoplasmic efflux, and overall collapse of the cytoskeleton, which intuitively corroborated the antibacterial mechanism previously described.

For special wounds like diabetic wounds, which are abundant in tissue exudate and nutrients like glucose, they are particularly susceptible to attack by pathogenic bacteria. In the context of bacterially infected diabetic wounds, dressings serving as temporary barriers to the skin are required not only to rapidly eradicate bacteria from the microenvironment of the wound, but also to provide continuous clean outer environments for wounds that are highly susceptible to bacterial parasitism, in order to shield the wound from the interference of bacterial growth. In this experiment, the GAPEU hydrogel was co‐incubated with gram‐negative and gram‐positive bacteria for 12, 24, 36, 48, 60, and 72 h, and the survival status of the bacteria was quantified by turbidimetry, thus evaluating the long‐term bacterial inhibition effect of the hydrogel (Figure 5K). Noticeably, the inhibitory efficiency of GAPEU hydrogel for *S. aureus*, *E. coli*, and *MRSA* was still up to 93.02%, 90.11%, and 91.52%, respectively, at 72 h of co‐culture. The above quantitative results indicate that the hydrogel possesses superior long‐term bacterial inhibition to construct continuous lines of defense for wounds. The construction of sustainable infection lines of defense not only prevents the formation of biofilms in wounds due to the growth of massive amounts of bacteria, but also reduces the frequency of dressing changes, thus relieving the patients' pain. The reason for the sustainable construction of infection lines of defense by hydrogels is that the rigid topological network structure formed by covalent bond cross‐linking can effectively lock the oligo‐polyethyleneimine in the system and release it gradually over prolonged periods, thereby continuously inhibiting bacterial growth.

In reality, inappropriate activities in bacteria‐rich environments for patients with chronic wounds can cause large amounts of bacteria to invade the wound. Moreover, irregular wound observation and medical interventions will inevitably lead to bacterial infiltration into the nascent tissue. This condition requires dressings with the capacity to repeatedly eliminate substantial quantities of bacteria. For this purpose, the repetitive bactericidal capacity of GAPEU hydrogel was assessed by cyclic bactericidal tests. The specific evaluation process is illustrated in Figure 5I. In this experiment, the same hydrogel was repeatedly placed in the fresh bacterial suspension with the same bacterial concentration for co‐culture for 12 h. The repetitive bactericidal rate of the hydrogel was then quantified by turbidimetry. When GAPEU hydrogel was used to treat fresh bacterial suspensions of *S. aureus*, *E. coli*, and *MRSA* for the third time, its bactericidal rates were maintained at 86.69%, 82.65%, and 83.36%, respectively. Notably, the GAPEU hydrogel exhibited no substantial reduction in the bactericidal rate of gram‐negative and gram‐positive bacteria during the three cycles of the bactericidal procedure (Figure 5L). The above results indicate that GAPEU hydrogel has excellent cyclic antimicrobial ability. The essential reason is also due to the ability of GAPEU hydrogel to release oligo‐polyethyleneimine slowly over a long period of time, which is consistent with the fundamental reason for the long‐term antibacterial ability of the hydrogel. Furthermore, combined with the long‐term antibacterial results and the repetitive bactericidal results of the hydrogel, it is clear that the oligo‐polyethyleneimine slowly overflows from the hydrogel system rather than being released explosively, which fully proves that the hydrogel system has excellent slow‐release capability. This not only continues to kill and inhibit bacterial growth and proliferation, creating a sustained line of defense against infection for chronic wound healing, but also avoids unnecessary damage to nascent granulation tissue from the initial burst of cationic antimicrobial release.

**Figure 6:**
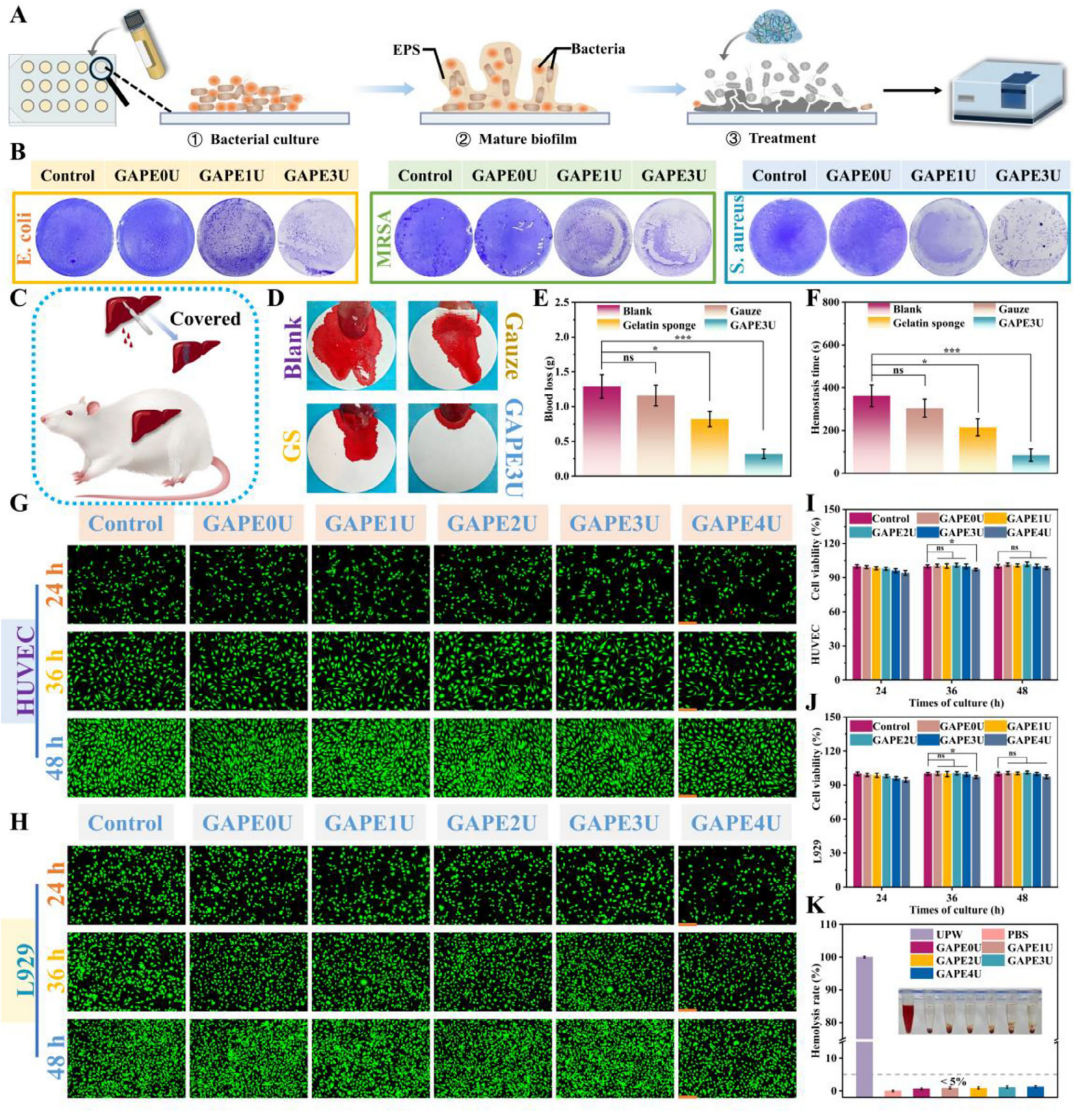
Antibiofilm properties and biocompatibility of hydrogels. A,B) Antibiofilm schematic and crystal violet staining, C–F) Hemostatic properties, G–J) Cytocompatibility test, K) Hemolysis test. Data are mean ± standard deviation (*n* ≥ 3); one‐way analysis of variance (ANOVA); P values: *P < 0.05, **P < 0.01, ***P < 0.001.

Delaying or inhibiting the development of bacterial resistance is at the heart of the response to the threat of the “post‐antibiotic era”. The development of bacterial resistance was evaluated by monitoring the ability of the GAPEU hydrogel to eliminate successive generations of *S. aureus*, *E. coli*, and *MRSA* (Figure 5D). Apparently, the antimicrobial capacity of the hydrogel did not show significant fluctuations, indicating that the GAPEU hydrogel did not develop resistance to the bacteria. This result is attributed to the fact that cationic antimicrobials work by affecting the permeability of bacterial cell membranes, disrupting their normal metabolism and leading to bacterial apoptosis. It does not result in genetic mutation of the bacteria to produce resistant dominant bacterial populations. In addition, the removal ability of GAPEU hydrogel for mature biofilms was evaluated (**Figure** **6**A,B). Statistically, it was found (Figure S13, Supporting Information) that the ability of GAPE3U hydrogel to remove mature biofilms of *E. coli*, *S. aureus*, and *MRSA* was 76.32%, 81.07%, and 78.64%, respectively.

### 2.11 Evaluation of Biocompatibility and In Vitro Hemostasis of Hydrogel

The dressing utilized for temporary skin should possess exceptional biocompatibility, which is of considerable significance for tissue regeneration. The biological application safety of GAPEU hydrogels was evaluated in this study by hemocompatibility and cytocompatibility. Initially, a series of hydrogels were tested in hemolysis assays with RBC suspensions under simulated physiological environmental conditions (Figure 6K). Notably, the ultrapure water‐treated group exhibited a vivid red coloration, indicative of substantial erythrocyte rupture. Conversely, a series of GAPEU hydrogel‐treated groups exhibited the color similar to the PBS‐treated group, thereby macroscopically demonstrating the excellent hemocompatibility of the GAPEU hydrogel. Subsequent quantitative analysis revealed that the hemolysis rate of the GAPEU hydrogel groups was significantly lower than that of the ultrapure water‐treated group, with rates of less than 5% for all groups, meeting the international standards for biomedical material application. The biosafety of the GAPEU hydrogel was further assessed by deepening the assessment with two important relevant cells in the tissue regeneration procedure (fibroblasts (L929) and venous endothelial cells (HUVEC)). Cells cultured with hydrogel extract were subjected to live‐dead staining at specific time points. As expected, the results demonstrated an increase in green fluorescence density, and the cells were dispersed uniformly attached to the wall, and the cell morphology was mostly spindle‐shaped, indicating favorable cell viability and robust growth (Figure 6G,H). Furthermore, the proliferative behavior of the cells was quantified by CCK‐8 assay (Figure 6I,J). After the 24 h co‐culture period with the hydrogel extract, it was observed that the proliferation viability of the cells in the experimental groups exhibited a slight decrease compared to that of the L929 cells in the blank group. However, the proliferation viability remained above 94.5% of that of the blank group in all groups. Furthermore, it is evident that the inhibitory effect on cells exhibits a slight increase as the content of oligo‐polyethyleneimine in the hydrogel system increases. Further co‐culture for 48 h revealed that the proliferation viabilities of the cells in the experimental groups were 100.67% (GAPE0U), 100.42% (GAPE1U), 101.17% (GAPE2U), 99.87% (GAPE3U), and 97.37% (GAPE4U), respectively, as compared to the blank group. Obviously, the cell proliferation behavior of the GAPE4U hydrogel‐treated group also showed a slight inhibitory situation, consistent with the live‐dead staining results described above. In view of this, after balanced assessment of the antimicrobial behavior and biocompatibility performance of GAPEU hydrogel, further bio‐functional exploration of GAPE4U hydrogel was abandoned. While the proliferation of cells in other experimental groups was not significantly different from the blank group. Upon comprehensive analysis of the above results, the GAPEU hydrogel is shown to exhibit favorable cytocompatibility.

According to in vitro adhesion tests, the GAPEU hydrogel demonstrates notable adhesion capabilities and can effectively seal skin and small intestine injury holes. The surface scratching of liver enriched with vascular network was further used to construct the rat liver hemorrhage model (Figure 6C). This model was utilized to evaluate the in vivo hemostatic behavior of GAPE3U hydrogel. As demonstrated in Figure 6D, compared with the bloodstain photographs of the blank, gauze‐treated, and gelatin sponge‐treated groups, the scratched livers treated with GAPE3U hydrogel exhibited only a few bloodstains. This finding intuitively illustrates the superior hemostatic ability of GAPE3U hydrogel. The study further quantified blood loss (Figure 6E) and hemostasis time (Figure 6F) for different treatments of scratched livers. The quantity of blood loss was 1.29 g (Blank), 1.16 g (Gauze), 0.82 g (Gelatin sponge), and 0.32 g (GAPE3U), whose hemostasis times were 362.24 s, 304.61 s, 214.33 s, and 85.36 s, respectively. Notably, the blood loss observed in the GAPE3U hydrogel‐treated group was ≈39.02% of that in the gelatin sponge‐treated group and only 27.58% of that in the gauze‐treated group. Furthermore, the hemostasis time of the scratched liver in the GAPE3U hydrogel‐treated group was reduced by 60.17% compared to the gelatin sponge‐treated group, and by 71.98% compared to the gauze‐treated group. Based on the above hemostasis time and blood loss data, it is clear that the emergency hemostatic ability of GAPE3U hydrogel for scratched liver is significantly better than that of gauze and gelatin sponge, which also quantifies the excellent in vivo hemostatic performance of GAPE3U hydrogel. The superior procoagulant efficacy of GAPE3U hydrogel in addressing scratched liver wounds can be attributed to several factors. i) The GAPE hydrogel exhibits the capacity to microfluidically adapt to irregular liver wounds, while its polar groups on the surface rapidly break down the barrier of the hydration layer on the liver surface. This process establishes extensive interactions with the liver wounds, such as hydrogen bonding, thereby creating the necessary conditions for interaction forces at the tissue‐hydrogel interface. Subsequent to UV intervention, covalent bond cross‐linking contributed to the formation of rigid topological network structure in the hydrogel, thereby significantly enhancing its cohesive energy. The above two aspects synergistically endow the hydrogel with outstanding adhesion properties, enabling it to provide strong sealing of bleeding wounds. ii) The polymer chains that constitute the hydrogel's topological network structure are abundant in amino groups, and they can interact with negatively charged platelets through electrostatic interaction forces to activate the coagulation cascade reaction. iii) The hydrogel rapidly absorbs tissue exudate and plasma from liver scratch wounds and locally enriches blood cells (erythrocytes and platelets, etc.) and coagulation factors through the concentration effect, thereby accelerating the coagulation cascade reaction in the wound.

### 2.12 Evaluation of Wound Healing Efficacy of Hydrogel in an Infection Model of Athletic Skin Defects

**Figure 7:**
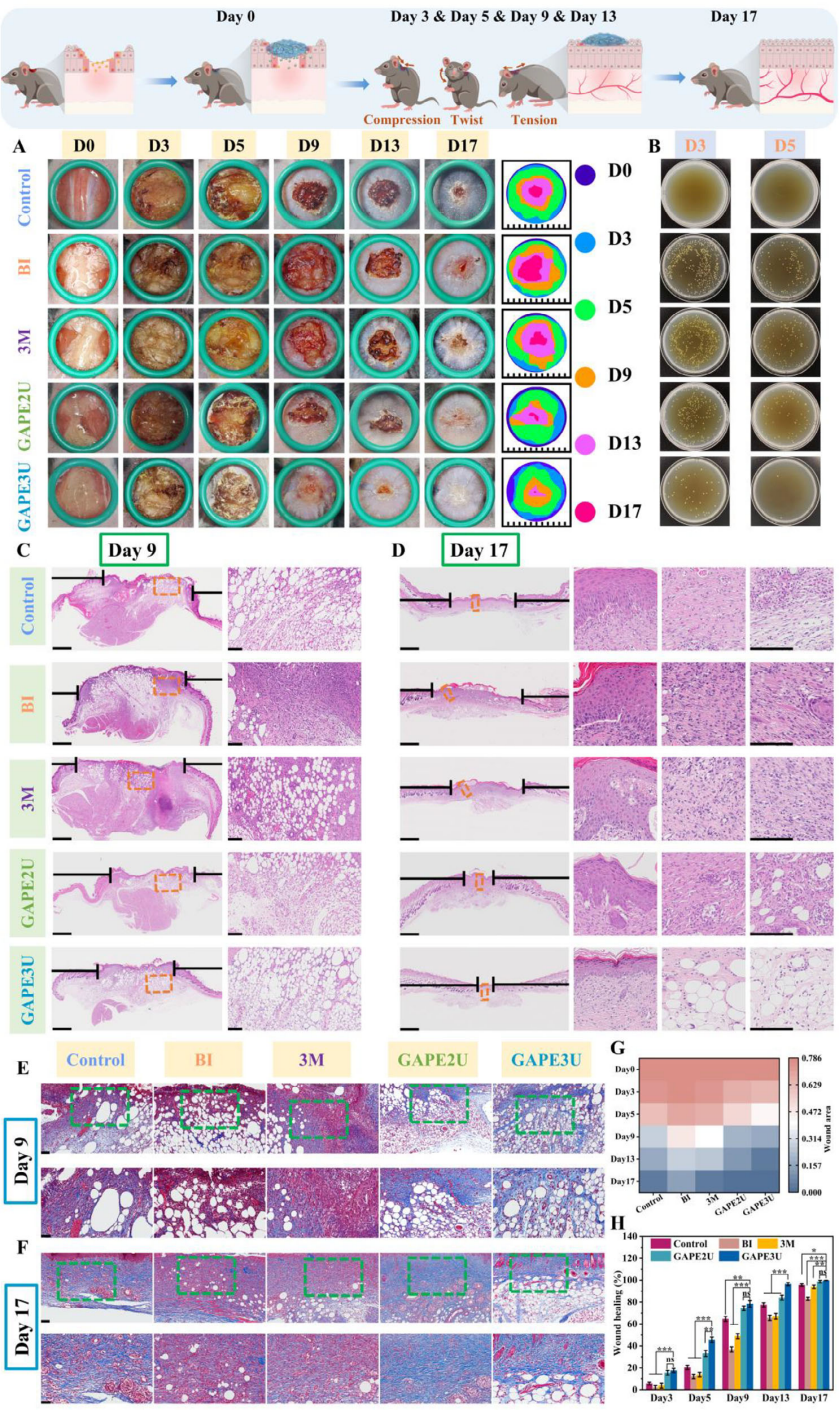
Macrograph of wound healing results and histologic analysis. A,G,H) Macrogram of wound and statistical analysis, B) In vivo antimicrobial results, C,D) H&E staining (scale bar of overall image: 2.5 mm, scale bar of enlarged image: 100 µm), E,F) Masson staining (scale bar of overall image: 100 µm, scale bar of enlarged image: 50 µm). Data are mean ± standard deviation (*n* ≥ 3); one‐way analysis of variance (ANOVA); P values: *P < 0.05, **P < 0.01, ***P < 0.001.

Based on the above tests and evaluations, GAPEU hydrogel exhibits excellent mechanical, injectable, microfluidic, self‐healing, and adhesion properties, along with painless removal from skin tissue. In particular, hydrogels demonstrate low energy dissipation, which can convert most of the energy imparted by the tensile external force into elastic potential energy stored in the topological network, thus contributing to hydrogels exhibiting strong retraction force. These findings indicate that hydrogels have significant potential for use in special biological tissue repair. In such instances, chronic wounds at sites of movement can result in stress concentrations at the wound edges due to unavoidable pulling. This phenomenon not only results in recurrent bleeding, thereby increasing the risk of infection, but also leads to abnormal procedures for cell proliferation and re‐vascularization. In this study, the nape of mice (the site where twisting, pulling, and compression occur at high frequencies) was selected to establish chronic infected wound models at the movement sites by repeated infections with massive amounts of *MRSA*. These mice were subjected to different treatments, and thus the regulation of the wound inflammation‐proliferation‐remodeling program by the GAPEU hydrogel could be compared and evaluated (**Figure** **7**A). Based on the characteristics of difficult‐to‐heal wounds in joint areas, and in accordance with the basic principles of observing inflammatory responses in the early stage, proliferative results in the middle stage, and the remodeling of new tissue in the late stage, we selected the 3rd, 5th, 9th, 13th, and 17th days to take photographs or collect samples to track the healing progress of wounds treated with different methods. It is evident from the optical picture of wound healing on day 3 that the scabs of the wounds on the neck dorsum were all ruptured, and rebleeding of the wounds was observed. This finding visually illustrates that the wounds suffered from frequent motion disturbances, thereby demonstrating the successful establishment of the motion wound model. Upon comprehensive analysis of the wound healing optical pictures, the wound in the GAPEU hydrogel‐treated group exhibited a superior regeneration rate compared to the other groups. Especially, the GAPE3U hydrogel‐treated group demonstrated significantly superior healing outcomes compared to the other groups at day 5. This phenomenon can be attributed to the ability of GAPEU hydrogels to rapidly eradicate bacteria from the wound, thereby slowing down and harmonizing the wound inflammatory response. Furthermore, they provide long‐term antibacterial action to maintain a clean healing microenvironment for the wound. Subsequently, bacterial colonization of wounds in different treatment groups was quantified using the spread plate method. When the wound healed to day 3 (Figure 7B), the GAPEU hydrogel‐treated group had extremely few bacterial colonies compared to the infected + PBS‐treated wound group and the infected + commercial 3M hydrogel‐treated group, directly confirming the excellent in vivo antibacterial capacity of the hydrogel. Subsequent follow‐up revealed that the number of bacterial colonies in the GAPEU hydrogel‐treated group was reduced further compared to the other groups when healing proceeded to the fifth day, which fully demonstrated the excellent long‐term antibacterial ability of the hydrogel. Furthermore, the process of wound healing was quantitatively tracked for each group by identifying and calculating the unhealed area of each group's wounds at different time points (Figure 7G). The corresponding wound healing rate for each group was then calculated (Figure 7H). Notably, at day 9, the area of unhealed wounds was significantly lower in the GAPE2U hydrogel‐treated group (74.39%) and the GAPE3U hydrogel‐treated group (78.34%) than in the group with non‐infected + PBS treatment (64.59%), the group with infected + PBS treatment (36.82%), and the group with infected + commercially available 3M hydrogel treatment (48.92%). As the healing procedure progressed (on the 17th day), the immune systems of the infected wounds gradually recovered, and the absent skin in the non‐infected + PBS‐treated group, the infected + PBS‐treated group, and the infected + commercial 3M hydrogel‐treated group also showed a certain degree of regeneration. However, at this time, the wounds in the GAPE2U hydrogel‐treated and GAPE3U hydrogel‐treated groups exhibited nearly complete healing, in contrast to the wounds in the non‐infected + PBS‐treated, infected + PBS‐treated, and infected + commercialized 3M hydrogel‐treated groups, which showed significant unhealed areas of 4.46%, 16.94%, and 5.99%, respectively. The above results show that the commercial 3M hydrogel treatment group and the blank control group exhibit severe bacterial colonization with a slow wound healing process. In contrast, the “energy transit station” hydrogel can establish an infection defense line for the wound, regulate the inflammatory response, and promote its rapid transition to the proliferation‐remodeling stage.

### 2.13 Histologic Evaluation of Regenerated Tissue from Infected Sports Wounds

Chronic bacterial infections in wounds undergo inflammatory, proliferative, and remodeling procedures in the spiral, progressive, and sequential manner to accomplish regeneration of the defective tissue.^[^ ^21^ ^]^ In this study, to thoroughly analyze the overall harmonizing effect of GAPEU hydrogel on chronic sports wounds, hematoxylin and eosin (H&E) staining and Masson staining were utilized to assess the inflammatory response of the wounds and the quality of regenerated tissues in the refined manner. As demonstrated in the H&E staining of the nascent tissues on day 9 (Figure 7C), the wounds in the infected + PBS‐treated group and the infected + commercial 3M hydrogel‐treated group exhibited loose nascent tissues accompanied by substantial infiltration of inflammatory cells, suggesting strong inflammatory response. Furthermore, there is a notable accumulation of inflammatory cells such as neutrophils and macrophages in the subcutaneous muscle tissue. This finding indicates that the bacteria have spread unchecked at the wound tissue, resulting in the infection penetrating deep into the muscle tissue, which is consistent with the in vivo antibacterial results. In contrast, the wounds in the GAPE2U hydrogel‐treated group and the GAPE3U hydrogel‐treated group did not exhibit abnormal inflammatory responses, indicating that the GAPEU hydrogel could rapidly remove bacteria from the wounds and effectively alleviate the inflammatory cascade reaction in the infected defective skin tissues. After treatment for 17 days (Figure 7D), H&E staining revealed that the regenerated epidermis structure was heterogeneous in the infected + PBS‐treated and infected + commercial 3M hydrogel‐treated groups. Their scar widths were 5.04 and 3.62 mm, respectively, which were significantly higher than those of the non‐infected + PBS‐treated group (3.28 mm), the infected + GAPE2U hydrogel‐treated group (1.24 mm), and the infected + GAPE3U hydrogel‐treated group (0.58 mm). Significant indices, including epidermal thickness, granulation tissue thickness, and the degree of hair follicle formation, were used to quantify the progression of wound proliferation and remodeling. After statistical analysis (Figure S19C, Supporting Information), the thickness of granulation tissue was 0.65, 0.93, 0.71, 0.57, and 0.25 mm in the non‐infected + PBS‐treated group, infected + PBS‐treated group, infected + commercial 3M hydrogel‐treated group, infected + GAPE2U hydrogel‐treated group and infected + GAPE3U hydrogel‐treated group, respectively. Apparently, GAPEU hydrogel‐treated wounds exhibited more perfect thin granulation tissue. Furthermore, (Figure S19D, Supporting Information) the epidermal thickness of wounds in the infected + GAPE2U hydrogel‐treated group and infected + GAPE3U hydrogel‐treated group was 108.87 and 49.41 µm, respectively, which were remarkably thinner than that of the non‐infected + PBS‐treated group (138.22 µm), infected + PBS‐treated group (227.03 µm), and infected + commercial 3M hydrogel‐treated group (174.85 µm). During the re‐epithelialization process of the wound, the thicker granulation tissue and epidermis are gradually transformed into mature thin granulation tissue and epidermis. The comparative analysis of the granulation tissue and epidermis in each group revealed that wounds in the infected + GAPEU hydrogel‐treated group showed more rapid reparative progress and had already entered the early stages of the remodeling phase. Moreover, the hair follicles (significant dermal appendages) of the wounds in the infected + GAPE2U hydrogel‐treated group and infected + GAPE3U hydrogel‐treated group exhibited superior regeneration (Figure S19E, Supporting Information). The results clearly demonstrate that the wounds in the GAPEU hydrogel‐treated group were already in the remodeling phase, consistent with the conclusions presented above for epidermal thickness and granulation tissue thickness.

Furthermore, the collagen deposition and alignment of wounds in each group was assessed with Masson staining. Notably, upon healing to day 9 (Figure S19A, Supporting Information), the collagen deposition density of the wounds in the infected + GAPE2U hydrogel‐treated group and infected + GAPE3U hydrogel‐treated group was 59.03% and 65.89%, respectively, which was significantly better than that of the non‐infected + PBS‐treated group (32.95%), infected + PBS‐treated group (20.18%), and infected + commercial 3M hydrogel‐treated group (27.22%). The above results clearly illustrate that the infected wounds presented more excellent collagen deposition after GAPEU hydrogel treatment, which would facilitate the reconstruction of the extracellular matrix structure. To further analyze the extracellular matrix reconstruction in the wounds of different treatment groups, Masson staining was performed on the wounds of each group whose healing was progressing up to day 17 (Figure 7F). In comparison to the wounds in the non‐infected + PBS‐treated group, the infected + PBS‐treated group, and the infected + commercial 3M hydrogel‐treated group, the collagen of wounds in the infected + GAPEU hydrogel‐treated group exhibited a transformation from disorganized depositional state to dense and well‐ordered bundles of collagen, further interlocking to form topologically‐structured collagen networks. The above results sufficiently illustrate that the wounds in the infected + GAPEU hydrogel‐treated group showed more excellent extracellular matrix reconstruction, which would facilitate cell adhesion, proliferation, and differentiation.

As can be seen from the above results, inflammatory cells in the wounds of the commercial 3M hydrogel treatment group and the blank control group are widely aggregated in the subcutaneous muscle tissue, indicating that bacteria have spread uncontrollably in the wound tissue. In contrast, the wounds in the “energy transit station” hydrogel treatment group did not show abnormal inflammatory responses. Meanwhile, the wounds in the commercial 3M hydrogel treatment group and the blank control group exhibited wider scars, thicker epidermis and granulation tissue, and disordered collagen deposition and reorganization. However, the extracellular matrix, skin and its appendages in the wounds of the “energy transit station” hydrogel treatment group showed better regeneration quality.

### 2.14 Assessment of Inflammatory Response and Macrophage Polarization in Regenerating Tissues of Infected Sports Wounds

**Figure 8:**
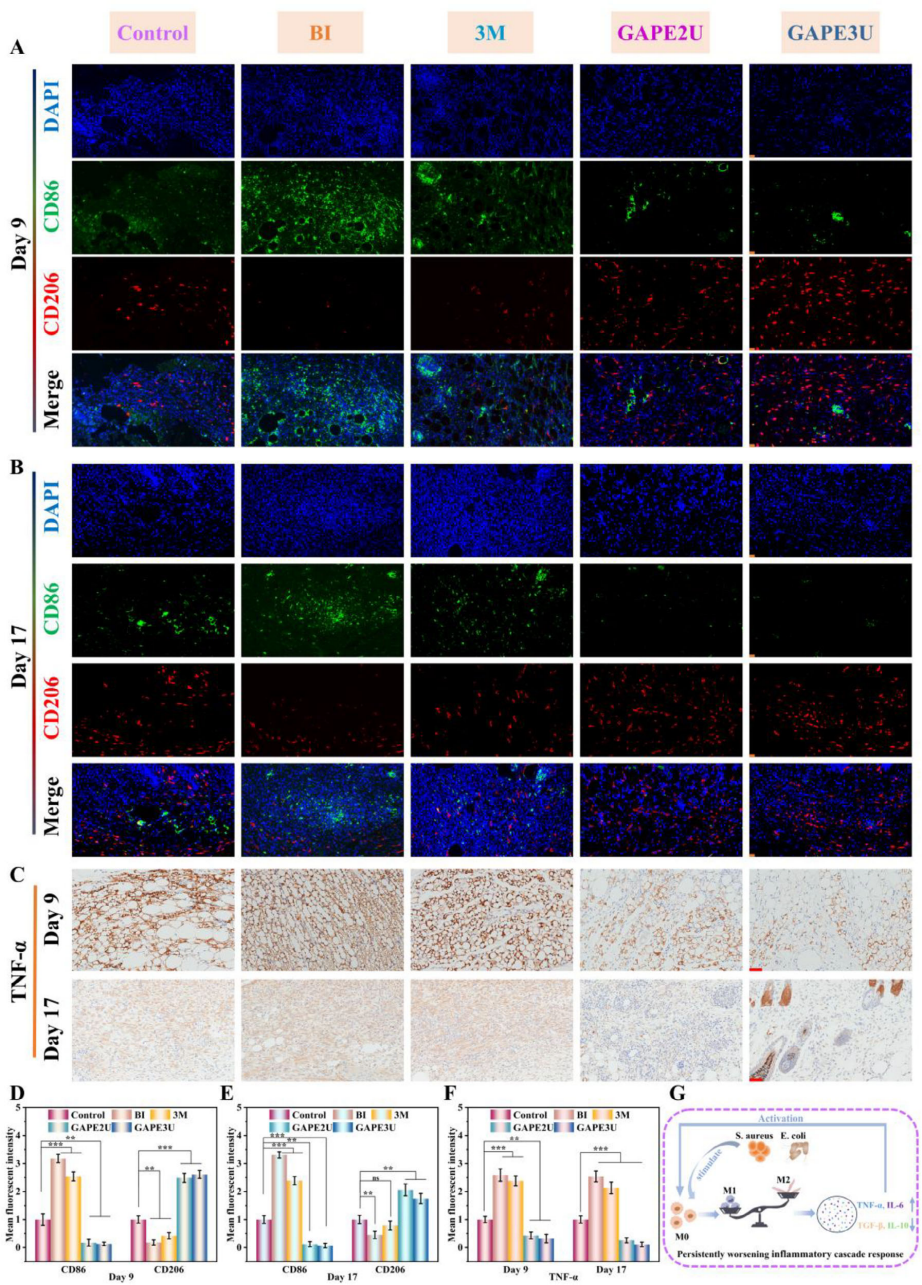
Macrophage phenotype and inflammatory factor distribution. A,B,D,E) Macrophage phenotype and statistics (scale bar: 20 µm), C,F) TNF‐α distribution and statistics (scale bar: 50 µm), G) Malignant diagram of the inflammatory cascade response. Data are mean ± standard deviation (*n* ≥ 3); one‐way analysis of variance (ANOVA); P values: *P < 0.05, **P < 0.01, ***P < 0.001.

Chronic infected wounds that are difficult to heal are characterized by repeated bacterial colonization and growth, resulting in the disturbed inflammatory response.^[^ ^4^, ^22^ ^]^ Consequently, the wound is infiltrated with high levels of inflammatory cells and inflammatory factors for an extended period of time. To further analyze the inflammatory response of the wounds in each treatment group, qualitative and quantitative analyses of inflammatory factors and inflammatory cells in the wounds were performed using immunohistochemistry and immunofluorescence staining methods. On the ninth day of healing (**Figure** **8**C,F), wounds in the infected + PBS‐treated and infected + commercial 3M hydrogel‐treated groups exhibited high levels of the proinflammatory factor TNF‐α. Conversely, wounds in the infected + GAPEU hydrogel‐treated group displayed low levels of the proinflammatory factor TNF‐α. Despite healing to day 17, wounds in the infected + PBS‐treated and infected + commercial 3M hydrogel‐treated groups exhibited higher concentrations of the pro‐inflammatory factor TNF‐α. This finding indicates that the capacity of the GAPEU hydrogel to rapidly eliminate bacteria from wounds can potently mitigate bacterial stimulation of the immune system in wounds, thereby coordinating the infiltration of inflammatory factors. The inflammatory cells (macrophages) of wounds were subsequently subjected to qualitative and quantitative analysis. Macrophages are vital for the inflammatory response and tissue regeneration in wounds. Specifically, M1‐type macrophages recruit inflammatory cells by releasing pro‐inflammatory factors (including TNF‐α and IL‐6) to rapidly eliminate bacteria.M2‐type macrophages coordinate cell proliferation and differentiation and extracellular matrix remodeling by secreting anti‐inflammatory factors (TGF‐β and IL‐10). As illustrated in Figure 8A,D, wounds in the non‐infected + PBS‐treated group, infected + PBS‐treated group, and infected + commercial 3M hydrogel‐treated group exhibited substantial M1‐type macrophage infiltration (CD86, green fluorescence) by day 9 of healing. However, the majority of macrophages in wounds in the infected + GAPEU hydrogel‐treated group exhibited the M2‐type (CD206, red fluorescence). The above results fully confirmed that the wounds in the infected + PBS‐treated and infected + commercial 3M hydrogel‐treated groups were still in the stage of severe inflammatory response. In contrast, the wounds in the GAPEU hydrogel‐treated group had already passed through the inflammatory stage and entered the proliferation‐remodeling phase. Subsequent analysis of immune cells from the wounds was conducted to assess the inflammatory cascade response of the wounds. It is noteworthy that healing to 17 days (Figure 8B,E), wounds in the infected + PBS‐treated group and infected + commercial 3M hydrogel‐treated group still have high levels of M1‐type macrophage infiltration. Combining the macrophage polarization and distribution on day 9, it is evident that massive bacterial colonization stimulates the immune response to recruit inflammatory cells, thus secreting extensive inflammatory factors. This results in the wound being in high concentrations of inflammatory cells and inflammatory factor infiltration. This will disrupt the normal inflammatory cascade reaction chain, causing the immune system to enter a state of temporary inflammatory cascade reaction disorder, resulting in the inflammatory reaction that enters a vicious cycle (Figure 8G). Consequently, this will lead to the wound remaining in the abnormal inflammatory reaction stage for an extended duration. Additionally, there was a downregulation of M2‐type macrophage density in the infected + GAPE3U hydrogel‐treated group as wounds healed to day 17. The analysis was further deepened in conjunction with the function of M2‐type macrophages. It was observed that the wounds in the GAPE3U hydrogel‐treated group had already passed the rapid proliferation phase of the cells, indicating that the infected wounds were in the early stage of the remodeling phase, which was consistent with the results presented by H&E staining.

### 2.15 Assessment of Vascular Regeneration in Regenerated Tissue from Infected Sports Wounds

**Figure 9:**
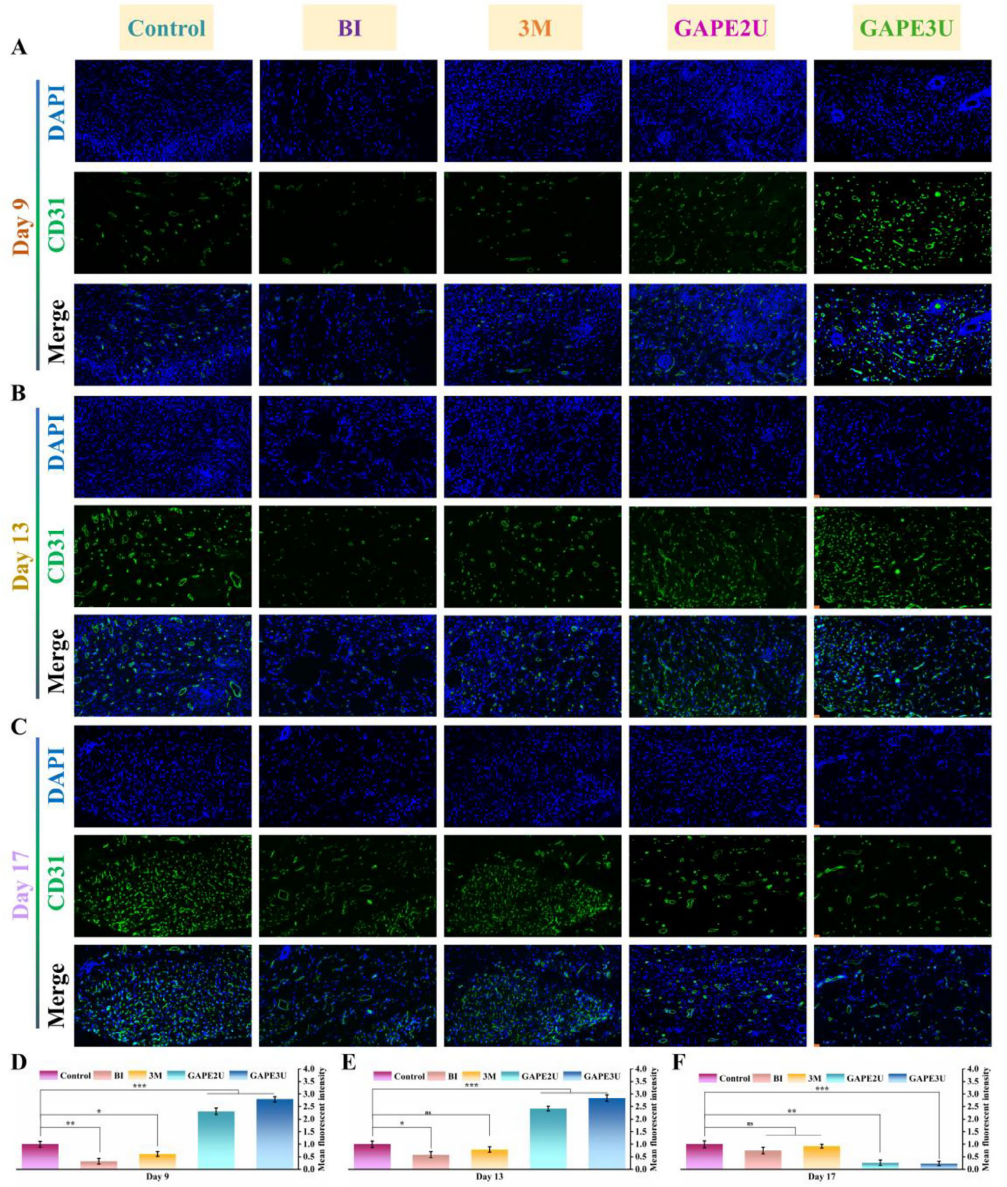
Distribution of the wound vascular network. A–C) CD31 immunofluorescence staining (scale bar: 20 µm), D–F) Vascular quantitative statistic graphs. Data are mean ± standard deviation (n ≥ 3); one‐way analysis of variance (ANOVA); P values: *P < 0.05, **P < 0.01, ***P < 0.001.

Vessels can not only provide the necessary oxygen and nutrients for the proliferation and regeneration of defective tissues, but also remove the waste products of cellular metabolism from wounds, so the construction of appropriate vascular networks is crucial for the proliferation and regeneration of traumatic tissues.^[^ ^23^ ^]^ In order to evaluate the specific effects of the GAPEU hydrogel on angiogenesis, in vitro migration and tubule formation experiments were first conducted on endothelial cells. The results are shown in Figures S16 and S17 (Supporting Information). Compared with the control group, the cell migration and tube formation abilities of the H_2_O_2_ group were significantly inhibited. In contrast, cell migration rates were significantly increased in the GAPE3U hydrogel‐treated group. At the same time, the GAPE3U hydrogel treatment group exhibited a denser vascular network, with a significant increase in the number of nodes. These results indicate that GAPEU hydrogel can effectively alleviate oxidative stress damage to cells and promote cell migration and angiogenesis. In addition, CD31 was utilized to fluorescently label the vessels in wounds at different time points, thereby assessing the remodeling status of the vascular network in the wounds. Notably, the wounds in the GAPEU hydrogel‐treated group exhibited superior vascular regeneration by day 9 (**Figure** **9**A,D), as compared to the non‐infected + PBS‐treated group, the infected + PBS‐treated group, and the infected + commercial 3M hydrogel‐treated group. Further tracking of the vascular reconstruction status of wounds in each treatment group indicated that the vascular network of wounds in the GAPEU hydrogel‐treated group exhibited increased density upon reaching day 13 of healing (Figure 9B,E). Upon detailed analysis of the above data, it was observed that the vascular reconstruction of wounds in the infected + PBS‐treated group was slow. This phenomenon may be attributed to severe infiltration of inflammatory factors and inflammatory cells by bacterial proliferation, resulting in the inflammatory cascade response becoming disordered and abnormal, thereby delaying the reconstruction of the extracellular matrix and vessels. This finding is consistent with the conclusions presented by H&E staining and macrophage polarization. Concurrently, the wounds in the GAPEU hydrogel‐treated group exhibited a rapid increase in vascularity, suggesting that the wounds were in the angiogenic subprogram of the wound proliferation phase at this time. Combined with the coordination mechanism of positive and negative feedback in the organism, it also indicates that the wounds of the GAPEU hydrogel‐treated group was in the highly active stage of cell proliferation and differentiation at this time.

While rapid remodeling of the vessels is important in the reconstructive procedures of defective skin, the unrestrained increase will inevitably lead to scar formation. Meanwhile, sports‐related wounds can cause stress concentrations at the wound edges due to frequent pulling. Excessive exogenous mechanical stimulation can also accelerate vascularization, cell proliferation, and collagen hyperplasia in the wound, resulting in abnormal scar formation. Therefore, it is crucial for the normal repair of the defective skin that sports wounds undergo vascular generation and reorganization procedures. In this study, the vascular network of the nascent tissue of wounds healed to 17 days was further tracked (Figure 9C,F). It can be found that the new blood vessels in the wounds of the commercial 3M hydrogel treatment group and the blank control group showed uncontrolled increase. Remarkably, in the GAPE2U hydrogel‐treated and GAPE3U hydrogel‐treated groups with excellent rebound properties, the vascular network of the wounds was reduced and remodeled. Combined with H&E staining and macrophage phenotype, it is evident that at this time, the wounds in the GAPEU hydrogel‐treated groups were in the remodeling phase, having passed the peak of cellular metabolism. The newborn tissues exhibited a decreased need for oxygen and nutrients, thus the organism reorganized the vascular network of the wounds through the feedback mechanism. This finding indicates that the probability of the wound ultimately forming scars is minimal.

### 2.16 Assessment of Edge Stress Situation of Regenerated Tissue from Infected Sports Wounds

**Figure 10:**
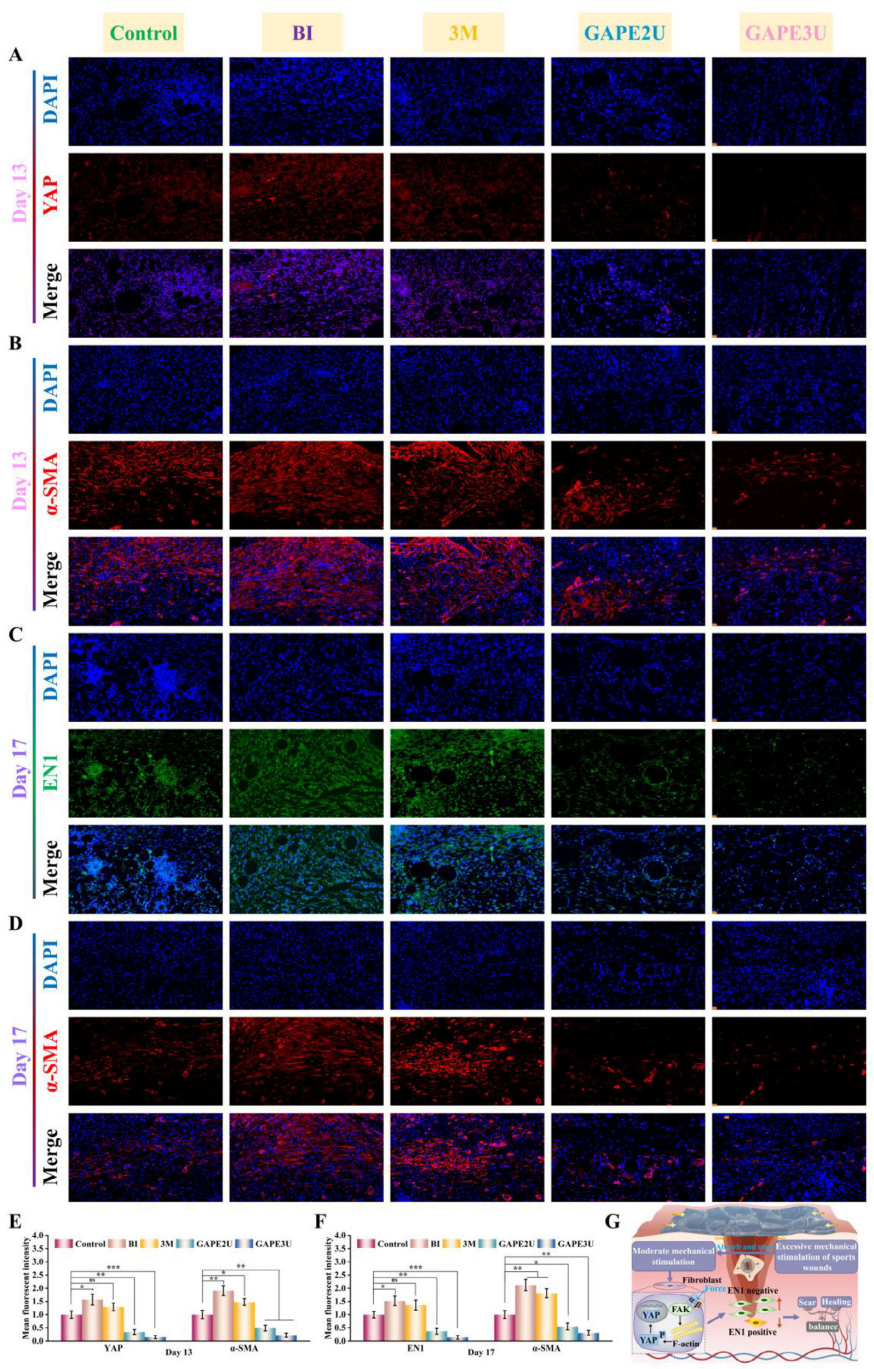
Wound edge stress analysis. A,E) YAP immunofluorescence and quantitative statistics (scale bar: 20 µm), B–F) α‐SMA immunofluorescence and quantitative statistics (scale bar: 20 µm), C,F) En1 immunofluorescence and quantitative statistics (scale bar: 20 µm), G) Diagram of the mechanism by which the hydrogel manages wound edge stress. Data are mean ± standard deviation (*n* ≥ 3); one‐way analysis of variance (ANOVA); P values: *P < 0.05, **P < 0.01, ***P < 0.001.

Combining the above animal in vivo experimental data, GAPEU hydrogel can rapidly remove bacteria colonized in the wound and inhibit bacteria for long term, thereby providing a clean microenvironment for wound healing. On this basis, it can relieve the stimulation of the immune system of the wound by bacteria, which in turn harmonizes the macrophage phenotype, thus shortening the inflammatory response of the wound and facilitating the successful entry of the wound into the proliferative repair phase. However, the constant motion of non‐static wounds can result in excessive traction on the wound, leading to stress concentrations and abnormal mechanical stimulation of the wound edges. This phenomenon not only results in recurrent bleeding during the pre‐wound phase but also induces aberrant manifestations such as excessive cell proliferation and angiogenesis in wounds during the proliferation‐remodeling phase. It is imperative that dressings not only effectively eliminate bacteria and harmonize macrophage polarization, but also dynamically program mechanical stress transference in the athletic wound. In this study, by labeling cellular mechanotransduction transcriptional coactivator (YAP, Yes‐related protein), the intervention of GAPEU hydrogel with excellent rebound properties for mechanical stimulation of the edges of sports wounds during the proliferation‐remodeling phase was evaluated. Abnormal mechanical stimulation of the wound induced nuclear translocation of Yes‐associated protein (YAP), particularly in fibroblast populations that are sensitive to mechanical stimulation, such that En1 lineage‐negative fibroblasts (ENF) were transformed into En1 lineage‐positive fibroblasts (EPF).^[^ ^7^ ^]^ En1 lineage‐positive fibroblasts (EPF) induce the activation of cells such as keratinocytes and endothelial cells through direct cell‐to‐cell contact and/or indirect activities such as paracrine to secrete and produce more vascular endothelial growth factor (VEGF).^[^ ^12^ ^]^ This regulates the continuous high rate of increase in vascularity of the wound and stimulates the positive and negative feedback coordination mechanisms of the organism, resulting in excessive proliferation of fibroblasts and excessive remodeling of vessels at the wound.^[^ ^24^ ^]^ As demonstrated in **Figure** **10**A, the infected + PBS‐treated group and the infected + commercial 3M hydrogel‐treated group exhibited the high density of YAP immunofluorescence, while the GAPEU hydrogel‐treated group exhibited only minimal YAP immunofluorescence. The fibroblast phenotype was further explored. It was observed (Figure 10C) that wounds in the infected + PBS‐treated and infected + commercial 3M hydrogel‐treated groups had more extensive En1‐lineage‐positive fibroblasts compared to the GAPEU hydrogel‐treated group. To refine the regulatory mechanism of the “energy transit station” hydrogel on mechanical signal transduction in sports joint wounds, we tracked focal adhesion kinase (Fak) (Figure S21, Supporting Information), transforming growth factor‐β1 (TGF‐β1) (Figure S22, Supporting Information), and connective tissue growth factor (CTGF) (Figure S23, Supporting Information) in the nascent wound tissue of different treatment groups through immunofluorescence staining. Clearly, the nascent tissue in the control group and the 3M hydrogel treatment group exhibits high‐density Fak immunofluorescence, TGF‐β1 immunofluorescence, and CTGF immunofluorescence. Combined with the results of wound vascular network remodeling, it can be seen that after cells in the nascent tissue of the control group and the 3M hydrogel treatment group perceive excessive mechanical stimulation, they overactivate focal adhesion kinase (the key intracellular “mechanical force‐biochemical signal” converter). This in turn promotes YAP to translocate from the cytoplasm into the nucleus, regulating the activation and function of En1 lineage‐positive fibroblasts. Meanwhile, overactivated En1 lineage‐positive fibroblasts and YAP synergistically regulate the release and activation of TGF‐β1, while the activated TGF‐β1 also mediates the upregulation of CTGF, amplifying the mechanical force‐induced abnormalities in repair (excessive proliferation of fibroblasts, excessive collagen deposition, and excessive angiogenesis). In contrast, the coordination mechanism of positive and negative feedback in mechanical signal transcription remains in the normal state in the GAPEU hydrogel‐treated group. This indicates that the “energy transit station” hydrogel can convert exogenous mechanical stimulation into elastic potential energy within the topological network, avoiding frequent stress concentration at the wound edges and preventing excessive mechanical signal transduction to nascent tissue. This will shield and regulate the exogenous mechanical stimulation of sports wounds, guiding the wound's proliferative phase through normal subprocesses such as vascularization and re‐epithelialization.

On the other hand, excessive traction dilation of the wound will stimulate the wound to upregulate trauma contractility, namely, massive proliferation of myofibroblasts.^[^ ^13^ ^]^ While α‐SMA‐positive myofibroblasts lead to excessive remodeling of the ECM, creating microenvironments conducive to vascular growth. In this study, the distribution of myofibroblasts was presented by labeling α‐smooth muscle actin (α‐SMA) to visualize the contractile force of the traumatic nascent tissue against external pulling. It is evident (Figure 10B,D) that the wounds in the GAPEU hydrogel‐treated groups exhibited a gradual decrease in α‐SMA fluorescence intensity with the progression of nascent tissue proliferation‐remodeling when compared to the wounds in the infected + PBS‐treated group and the infected + commercial 3M hydrogel‐treated group, which exhibited a high density of α‐SMA fluorescence intensity. The above results indicate that myofibroblasts in the wounds of the GAPEU hydrogel‐treated group gradually perish, directly reflecting the gradual decrease in the contractility of the wound and also suggesting that the wounds were not subjected to excessive exogenous mechanical stimulation. It strongly demonstrates that GAPEU hydrogel dressing can shield the wound from frequent mechanical stimulation from sports and ensure the wound undergoes a normal and rapid proliferation‐remodeling phase, avoiding the formation of proliferative scars with disorganized collagen fibers, active fibroblasts, and abundant vascular distribution.

Clearly, the nascent tissues in the control group and 3M hydrogel‐treated group are subjected to excessive mechanical stimulation during the healing process of sports wounds, overactivating factors related to mechanical‐fibrotic signal transduction, accompanied by the differentiation of specific fibrotic subpopulations such as myofibroblasts and En1 lineage‐positive fibroblasts, ultimately impairing the normal repair and regeneration of tissues. However, the GAPEU hydrogel can program the stress at the wound edges and regulate mechanical signal transduction in sports wounds through the exogenous pathway (YAP‐EPF pathway) and endogenous pathway (α‐SMA‐positive myofibroblasts) (Figure 10G), preventing moving wounds from entering the abnormal proliferation‐remodeling process due to inevitable frequent movements.

### 2.17 In Vivo Host Response

For biomaterials, the ability to demonstrate excellent compatibility within the in vivo environment is the critical prerequisite for their practical application. In view of this, GAPE3U hydrogel was implanted into the subcutaneous tissue of mice and the in vivo biocompatibility was evaluated by observing the host response to the material. After a period of 7 days, the skin tissue was analyzed by H&E staining (Figure S15, Supporting Information). The local area exhibited only a mild acute inflammatory reaction. Apparently, the GAPE3U hydrogel showed extremely low irritation to the skin tissue, indicating its excellent in vivo biocompatibility. Meanwhile, as can be seen from the H&E staining of organs (Figure S20, Supporting Information), 14 days after subcutaneous implantation of GAPE3U hydrogel into male C57 mice, no obvious histopathological abnormalities were observed in the major organs of the mice, including the heart, liver, spleen, lung, and kidney. This experimental result indicates that the potential risk of long‐term toxicity of GAPE3U hydrogel to the organs responsible for metabolism and excretion in mice is relatively low.

## 3 Conclusion

In summary, we constructed an elastic shrinkage hydrogel capable of harmonizing the inflammatory immune response to manage irregular chronic sports wounds infected with drug‐resistant bacteria. GAPEU hydrogel exhibits superior water retention and water supply capabilities to provide and maintain the moist healing microenvironment for wounds. Furthermore, it demonstrates favorable tensile and compressive properties, thus meeting the requirements for athletic wound repair. Meanwhile, in vitro bacterial evaluation tests showed that GAPEU hydrogel had excellent bacterial capture performance, long‐term antibacterial performance, repeated antibacterial performance, and antibiofilm performance, laying the foundation for rapidly eliminating bacteria from wounds. Moreover, as confirmed by in vivo antibacterial tests, GAPEU hydrogel has excellent bacterial management ability and can continuously build infection defense lines for wounds. As evident in in vivo tests, the GAPEU hydrogel exhibits remarkable bacterial management capabilities, effectively coordinating macrophage polarization at wounds, improving the infiltration situation of inflammatory factors and inflammatory cells in wounds, and promoting collagen deposition and vascular network construction during the early stages of wound healing. Notably, GAPEU hydrogel can absorb the marginal stress of the wound during the proliferation‐remodeling phase and convert it into elastic potential energy stored in the rigid molecular chains of the topological network, thereby shielding the athletic wound from frequent mechanical stimuli. Mechanotransduction of wounds was physically intervened and blocked to inhibit nuclear translocation of Yes‐associated protein (YAP) and to avoid the transformation of En1 lineage‐negative fibroblasts (ENF) into En1 lineage‐positive fibroblasts (EPF). Concurrently, GAPEU hydrogel down‐regulates the differentiation of fibroblasts into highly contractile α‐SMA‐positive myofibroblasts, thereby managing the nascent tissue proliferation‐remodeling phase that undergoes normal collagen deposition, vascular network formation, and reorganization. Overall, GAPEU hydrogel is a novel intelligent rebound wound dressing driven by elastic potential energy, providing a constructive and guided solution for managing the inflammatory cascade response and excessive exogenous mechanical stimulation of irregular sport wounds infected with resistant bacteria. However, we have not elaborated on the potential impact of individual age differences and different types of sports injuries on the research results, which may require detailed comparative analysis in subsequent studies.

## 4 Experimental Section

Preparation and characterization methods for hydrogels are detailed in the Supporting Information.

### Ethics Approval and Consent to Participate

All the animal experiments involved in this work were approved by the Ethics Committee of the First Affiliated Hospital of Naval Medical University (approval no. CHEC2025‐017).

## Conflict of Interest

The authors declare no conflict of interest.

## Supporting information



Supporting Information

## Data Availability

The data that support the findings of this study are available from the corresponding author upon reasonable request.
