# Supplementary material for: Mechanical Force Storage and Reprogramming Hydrogel for Scarless Repair of Sports Joint Wounds
Source: Adv Sci (Weinh). 2025 Sep 30;12(47):e11332. doi: 10.1002/advs.202511332 (PMC12713012; doi:10.1002/advs.202511332)
Supplement: Supplementary file 1 — Supporting Information [file ADVS-12-e11332-s001.docx]

**Mechanical Force Storage and Reprogramming Hydrogel for Scarless Repair of Sports Joint Wounds**

*Jiakang Zhang, Yuhui Zhang,* *Yuanru Lang, Tao Fu, Qian Liu, Long Chen,* *Peng Zhang,* *Yan Xiao, Yuntong Zhang ^⁎^, Shuo Fang ^⁎^,* *Meidong Lang ^⁎^*

Jiakang Zhang, Yuhui Zhang, Tao Fu, Qian Liu, Long Chen, Yan Xiao, Prof. Meidong Lang

Shanghai Key Laboratory of Advanced Polymeric Materials, Key Laboratory for Ultrafine Materials of Ministry of Education, School of Materials Science and Engineering, East China University of Science and Technology, 130 Meilong Road, Shanghai 200237, PR. China.

E-mail: mdlang@ecust.edu.cn

Yuanru Lang, Prof. Peng Zhang

Institute of Molecular Medicine, Renji Hospital, School of Medicine, Shanghai Jiao Tong University, Shanghai, 200127, China.

E-mail: pengzhang016@sjtu.edu.cn

Prof. Yuntong Zhang

Department of Trauma and Orthopedic Surgery, The First Affiliated Hospital of Navy Medical University, Shanghai, 200433 PR China.

E-mail: yuntong_zhang@smmu.edu.cn

Prof. Shuo Fang

Department of Plastic Surgery, The First Affiliated Hospital of Navy Medical University, Shanghai, 200433 PR China.

E-mail: fangshuo826@126.com

**1. Materials**

Type A gelatin (Gel, Bloom 221g), polyvinyl alcohol (type 1799), oligopolyethyleneimine (MW= 600 Da, 99%) were purchased from Shanghai Naicheng Biotechnology Co., Ltd. 5-Norbornene-2,3-Dicarboxylic Anhydride was obtained from Shanghai Dibo Chemicals Technology Co., Ltd. Triethylamine(TEA, ≥99.5%), 4-dimethylaminopyridine(DMAP, 99%) were bought from Shanghai Mindray Biochemical Technology Co., Ltd. 3-Aminophenylboronic acid (98%), 1-ethyl-(3-dimethylaminopropyl) carbodiimide hydrochloride (EDC-HCl, ≥99%), N-hydroxysuccinimide (NHS, 98%) were obtained from Shanghai New Platinum Chemical Technology Co., Ltd. Dulbecco's Modified Eagle Medium (DMEM), Cell Counting Kit-8 (CCK-8) were purchased from Shanghai Jiajun Biotechnology Co., Ltd. Fetal bovine serum (FBS), penicillin/streptomycin (P/S) were bought from Hangzhou Gaosheng Biotechnology Co., Ltd. Live/Dead Cell Toxicity Kit (calcein acetoxymethyl ester/propidium iodide) was obtained from Beijing InnoChem Science & Technology Co., Ltd. All chemicals are used directly to synthesize materials without further laboratory purification.

**2. Experimental section**

**2.1 Preparation of phenylboronic acid modified gelatin (Gel-APBA)**

The amidation reaction was utilized to modify 3-aminophenylboronic acid on A-type gelatin.^[1]^ The specific steps are as follows. Initially, 4 g of A-type gelatin (Gel) was dissolved in 200 mL of ultrapure water at 55°C under constant temperature stirring conditions. Following the complete dissolution of the gelatin, 3-aminophenylboronic acid was added to the system with continuous stirring until dissolution was complete. Subsequently, EDC-HCl and NHS were added sequentially as coupling agents, and the pH of the reaction system was adjusted to about 5.5 by hydrochloric acid aqueous solution. The reaction solution was subjected to continuous magnetic stirring at 55°C for 24 hours. Subsequent to the conclusion of the reaction, the reaction solution was transferred to dialysis bags with a molecular weight cut-off (MWCO) of 14,000 Da, and the solution was dialyzed with ultrapure water for five days. The Gel-APBA solid product was finally obtained by the lyophilization method. The qualitative analysis was conducted using the ^1^H NMR (AVANCE Ⅲ) and UV-Vis spectrometer (lambda 950). Concurrently, the standard curve was established based on the UV-Vis spectral data to realize the quantitative determination of the modified amount of 3-aminophenylboronic acid.

**2.2 Preparation of norbornene-modified polyvinyl alcohol (PVA-NB)**

Initially, 5 g of PVA (type 1799) was added to 100 mL of anhydrous DMSO, and the mixture was vacuumed under stirring conditions for 1 hour and passed through argon gas to remove air and water from the system. Thereafter, the mixture was heated to 90°C to ensure complete dissolution of PVA in anhydrous DMSO. The reaction system was cooled to room temperature and subjected to vacuum for 1 hour under stirring conditions, and argon was passed to ensure that the system was free of water and oxygen. Under the condition of passing argon gas, 5-norbornene-2,3-dicarboxylic anhydride, TEA, and DMAP were sequentially added to the reaction system. The system was subsequently placed in an inert environment under argon gas, and the reaction was carried out with continuous magnetic stirring at 50°C for 24 hours.^[2]^ At the end of the reaction, the reaction solution was transferred to dialysis bags with a molecular weight cut-off (MWCO) of 3500 Da and dialyzed with ultrapure water for 2 days. Subsequently, the solution was dialyzed overnight using the sodium carbonate buffer. The above operations are to be repeated twice. It was then dialyzed again for five days using ultrapure water. The norbornene-modified polyvinyl alcohol (PVA-NB) was obtained through the lyophilization treatment. The ^1^H NMR (AVANCE III) was utilized to characterize and quantify the PVA-NB molecule.

**2.3 Preparation of Gel-APBA/PVA-NB (GAPE/GAPEU) hydrogel**

Gel-APBA, oligo-polyethyleneimine, LAP initiator, and dithiothreitol were dissolved in ultrapure water to obtain a 6% (W/V) solution of Gel-APBA (containing different contents of oligo-polyethyleneimine, 0.2% LAP, and DTT). Furthermore, an 8% (W/V) solution of PVA-NB was prepared by thoroughly dissolving PVA-NB in ultrapure water. The polymer solutions were mixed in equal volumes, and GAPE hydrogels were prepared using a vortex oscillator. Further UV cross-linking treatment was performed to obtain GAPEU hydrogel. The oligo-polyethyleneimine content in the system was 0.00%, 0.45%, 0.70%, 0.95%, and 1.20%, respectively, based on which the hydrogels were named GAPE0U, GAPE1U, GAPE2U, GAPE3U, and GAPE4U, respectively.

**2.4 Characterization of basic properties of GAPE/GAPEU hydrogel**

**2.4.1 The gelation time of hydrogels**

The gelation time of GAPE hydrogels was determined using the inverted centrifuge tube method. The specific operations were as follows. The 6% (W/V) Gel-APBA solution and 8% (W/V) PVA-NB solution were transferred to the centrifuge tube at a volume ratio of 1:1 and mixed to form a homogeneous dispersion with the vortex oscillator. Subsequently, the tubes were inverted, and the end point of gelation was set at the point where the solution transformed into gel. The gelation time was recorded simultaneously. Each group of samples was tested eight times in parallel.

**2.4.2 Water retention properties of hydrogels**

In the open environment, the water retention property test was carried out on the same volume of hydrogel samples. The hydrogel samples were placed in clean petri dishes and left open at room temperature. The samples were weighed at two-hour intervals, and the water retention properties of the hydrogels were calculated in real time based on the following formula. Each group of hydrogel samples was tested seven times in parallel.

WR (%) = (W_n_/W_0_) × 100%

where W_0_ is the initial weight (g) of the hydrogel and W_n_ is the real-time weight (g) of the hydrogel at n hours.

**2.4.3 Water supply properties of hydrogels**

Experiments were conducted in an open environment to evaluate the water supply capacity of hydrogels.^[3]^ Simulated skin was initially prepared using the 20% gelatin aqueous solution. The hydrogel samples with identical specifications were uniformly fitted on the surface of the simulated skin, and the weight of the simulated skin was accurately weighed at two-hour intervals. The water supply properties of the hydrogel were calculated by the following formula. Each group of hydrogel samples was tested eight times in parallel.

WS (%) = (W_n_ - W_0_)/W_0_ × 100%

where W_0_ is the initial weight (g) of the simulated skin and W_n_ is the real-time weight (g) of the simulated skin after the hydrogel has been in contact with the simulated skin for n hours.

**2.4.4 Swelling properties of hydrogels**

The swelling properties of hydrogels were evaluated using gravimetric analysis.^[4]^ The lyophilized hydrogel of uniform volume was placed into centrifuge tubes with appropriate amount of PBS (pH=7.4, 37°C). The samples were then removed at predetermined time points, and the residual liquid was wiped from the surface with filter paper. The real-time weight of the hydrogel was then weighed and recorded until equilibrium was achieved. Each group of hydrogels was retested ten times. The swelling rate (SR) and equilibrium swelling rate (ESR) were calculated by the following formula:

SR= (W_t_ - W_0_)/W_0_

ESR= (W_eq_ - W_0_)/W_0_

where W_0_ is the initial dry weight (g) of the lyophilized hydrogel, W_t_ is the wet weight (g) after t hours of swelling, and W_eq_ is the wet weight (g) at the equilibrium of swelling.

**2.4.5 Degradation properties of hydrogels**

The degradation properties of hydrogels were assessed by gravimetric method.^[5]^ Lyophilized hydrogels of uniform size were accurately weighed for initial dry weight and placed in centrifuge tubes containing PBS (pH=7.4). Subsequently, the centrifuge tubes were placed in a thermostatic shaking incubator (37°C, 150 rpm), and the buffer was replaced with fresh buffer every 24 hours. The samples were removed at predetermined time points, rinsed thrice with ultrapure water to eliminate residual salts, and then dried to constant weight using the freeze dryer to calculate the degradation rate of the hydrogels. Each group of samples was tested eight times in parallel, and the degradation properties were calculated using the following formula:

Remaining weight (%) = (W_t_/W_0_) × 100%

where W_0_ is the initial lyophilized weight (g) of the hydrogel and W_t_ is the lyophilized weight (g) after t days of degradation.

**2.4.6 Topological morphology of hydrogels**

The scanning electron microscope (SEM, S-3400N) was utilized to observe and analyze the micro-morphological characteristics of the hydrogels. The hydrogel samples were subjected to swelling in PBS (pH=7.4, 37°C) until reaching equilibrium. Thereafter, the samples were removed and gently rinsed three times with ultrapure water to ensure the removal of residual salts from the surface. Subsequently, the samples were dried in a freeze dryer to constant weight. The surface of the lyophilized samples was sprayed with gold using an ion sputtering apparatus, and the cross-sectional morphology was observed and images were captured by SEM.^[6]^ Each group of hydrogels was prepared at least three independent samples, and five different field of view areas were randomly selected for each sample to be photographed. In total, at least 15 SEM images were acquired for each group of hydrogels. Subsequently, the pore sizes were counted and analyzed.

**2.5 Viscoelastic behavior and mechanical properties of hydrogels**

**2.5.1 Tensile testing of hydrogels**

A series of GAPEU hydrogels were prepared as rectangular sample strips of 3 cm in length and 1 cm in width. The mechanical properties (tensile strength and breaking elongation) of a series of hydrogels were evaluated using the uniaxial tensile program of the universal testing machine at a tensile rate of 20 mm/min. Each group of hydrogels was tested more than 3 times. The toughness of GAPEU hydrogels was calculated from the tensile curves.

**2.5.2 Compression testing of hydrogels**

The mechanical properties of GAPEU hydrogels were evaluated by single cycle compression test. Initially, the hydrogels were fabricated into cylindrical shapes with heights of 11.5 mm and diameters of 8 mm. Subsequently, the samples were placed between parallel compression plates of a universal testing machine (Shanghai Heng yi HY-0230) and compressed axially in cycles with a constant rate of 40 mm/min. The compressive strain of the GAPEU hydrogel single cycle compression test was set at 80%. Each group of hydrogel samples was tested three times in parallel. The compressive strength, toughness, and energy dissipation of the hydrogels were quantitatively evaluated from the 80% compressive stress-strain curve.

**2.5.3 Rheological properties of hydrogels**

Multi-modal testing of viscoelasticity and stability of hydrogels was carried out by rotational rheometer model MCR 501,^[7]^ which was implemented through the following three procedures:

(1) Dynamic strain sweep test. Strain sweep was performed on GAPE hydrogel and GAPEU hydrogel at a constant scanning frequency of 1 Hz. The linear viscoelastic region (LVR) of the hydrogels was determined from the simultaneous acquisition of energy storage modulus (G') and loss modulus (G'') curves. Each group of samples was tested three times in parallel.

(2) Dynamic frequency sweep test. Frequency sweeps (0.1 Hz -10 Hz) were conducted on the GAPE hydrogel by selecting 1% as the constant strain amplitude. The viscoelastic behavior of hydrogels was evaluated by comparing the numerical relationship between the low and high frequency bands G' and G''. The test was repeated 3 times for each group of samples.

(3) Time sweep test. Setting the test temperature to 25°C or 37°C (simulating room temperature and physiological environment), applying 1% constant strain and 1 Hz constant frequency, the GAPEU hydrogel was continuously scanned for 600 s. The stability of the hydrogels was evaluated by monitoring the magnitude of fluctuations in the energy storage modulus (G'') and loss modulus (G') over time. Each group of samples was tested three times in parallel.

**2.6 Evaluation of fatigue resistance and energy storage stability of hydrogels**

**2.6.1 Cyclic compression testing of hydrogels**

The fatigue resistance of GAPEU hydrogels was evaluated using the 100-cycle compression test.^[8]^ Cylindrical hydrogel samples were prepared according to the above compression test requirements. The surface of the hydrogel was sealed with silicone oil to minimize the influence of the external environment on the test results. Subsequently, the mechanical properties of the hydrogel at 80% compressive strain were evaluated by 100-cycle compression program of a universal testing machine at a rate of 40 mm/min. The time-stress performance of the GAPEU hydrogels was also finely evaluated based on the 100-cycle compression curves. The test was repeated four times for each group of hydrogels.

**2.6.2 Static compression testing of hydrogels**

The hydrogels were prepared in cylindrical shape as required for the compression test. The compressive strain of 80% was applied to the hydrogel between the compression plates at the constant rate of 40 mm/min. Subsequently, the compression plate was brought to rest, and the stresses on the GAPEU hydrogel were uninterruptedly monitored to assess the changes in the rebound stress (elastic recoil capacity) of the hydrogel over time. The stress relaxation behavior, energy dissipation behavior, and elastic potential energy storage capacity of GAPEU hydrogels during compression were evaluated. The test was repeated four times for each group of hydrogels.

**2.6.3 Static tensile testing of hydrogels**

A series of GAPEU hydrogels were prepared into rectangular hydrogels according to the uniaxial tensile test. The hydrogels were subjected to a tensile strain of 60% using a universal stretching machine at a rate of 20 mm/min. Upon reaching the tensile elongation of 60%, the stretching of the hydrogel sample was halted, and the stress changes in the hydrogel were continuously monitored. Thereby, the changes in tensile stress of GAPEU hydrogels with constant tensile rate were evaluated over time. The test was repeated four times for each group of hydrogels.

**2.7 Self-healing properties of hydrogels**

The hydrogel discs were initially stained with different dyes. Subsequently, the hydrogel discs were bisected along their central axis with a scalpel, and the cutting surfaces of the half discs of different colors were tightly fitted together. After a period of time, a bi-directional tensile force was applied to the repaired hydrogel discs to evaluate the self-healing effect of the hydrogel.

The quantification of the self-healing efficiency of GAPEU hydrogels was conducted by uniaxial tensile testing. Initially, the hydrogels were prepared into rectangular samples according to the uniaxial tensile test requirements previously delineated. A series of GAPEU hydrogels were cut transversely and symmetrically with the scalpel, and the cut surfaces were then tightly fitted and placed in the warm and humid environment to stay in place for 24 hours. The complete self-healing GAPEU hydrogels were subsequently subjected to uniaxial tensile testing (tensile rate of 20 mm/min). The test was repeated three times for each group of hydrogels. The tensile strengths of the original GAPEU hydrogels and the self-healing GAPEU hydrogels were recorded, and the self-healing efficiency of the hydrogels was calculated according to the following formula:

Self-healing efficiency (%) = (σ_self-healing_/σ_original_) × 100%

where σ_self-healing_ is the tensile breaking stress of the hydrogel after self-healing and σ_original_ is the tensile breaking stress of the original hydrogel.

**2.8 Injectable properties of hydrogels**

The 6% (W/V) Gel-APBA solution and the 8% (W/V) PVA-NB solution were placed in separate syringes and pushed synchronously through the dual-chamber syringe at a 1:1 volume flow ratio. The extrusion smoothness and moldability of the fluid during the mixing process were observed to macroscopically determine the injectability of the GAPE hydrogel. Shear rheology tests were subsequently performed using a rotational rheometer. The hydrogels were subjected to shear rate sweeps (0.01 s⁻¹-100 s⁻¹) at a constant temperature of 25°C. The shear-thinning behavior of GAPE hydrogels was evaluated by monitoring the change in hydrogel viscosity with shear rate.

**2.9 Micromobility properties of hydrogels**

The appropriate amount of GAPE hydrogel was placed in the beaker with glass beads, and then the beaker was moved to the constant temperature of 37°C. The data were tracked with the digital camera. The focal point is to assess the hydrogel's capacity for adaptively filling complex and irregular shapes. Subsequently, the hydrogels were exposed to UV light, resulting in the transformation of the GAPE hydrogels into GAPEU hydrogels. A comparative and analytical investigation was conducted on the microfluidity properties of GAPE and GAPEU hydrogels.

**2.10 Adhesion properties of hydrogels**

**2.10.1 Lap shear tensile test**

The porcine skin was initially cleansed and meticulously cut into rectangular pieces of 9 cm length and 2 cm width. The 6% (W/V) Gel-APBA solution was applied evenly to the surface of the porcine skin, while the 8% (W/V) PVA-NB solution was applied synchronously to the other piece. Subsequently, two rectangular pieces of porcine skin were affixed with their coated surfaces facing each other and irradiated with UV to ensure complete hardening for molding. The hydrogel was left at room temperature for 10 minutes to ensure complete adhesion to the porcine skin. The effective adhesion area between the two layers of porcine skin was ensured to be 2 x 1 cm^2^. The axial tensile test was conducted on adhesive porcine skins with the aid of a universal testing machine (Shanghai Heng yi HY-0230) at a tensile rate of 10 mm/min. The test was repeated more than three times for all hydrogel groups.

**2.10.2 Burst pressure test**

The real-time blast pressure monitoring system was first constructed (Figure S9B).^[9]^ After the porcine skin and small intestine tissues were cleaned, full-layer penetrating injury holes were prepared at the designated positions with the circular perforator of 2 mm diameter. The appropriate amount of GAPE hydrogel was applied to cover the surface of the injury holes of the wet state for blocking, and was left at room temperature for 10 minutes, which prompted the micro-flow of the hydrogel to adapt itself to the irregular wounds. Subsequently, strongly cross-linked GAPEU hydrogels were formed by further UV irradiation. PBS was then injected into the system at the constant flow rate of 2 mL/min and pressure changes were continuously monitored. The threshold pressure value at which the blockage fails is considered to be the adhesion capacity of the hydrogel. Each group of hydrogels should complete a minimum of three parallel tests.

**2.11 On-demand removal properties of hydrogels**

**2.11.1 Lap shear tensile tests**

Referring to the previous lap shear tensile test, the porcine skins were initially cut into rectangular pieces and completed with hydrogel adhesion pretreatment. Appropriate quantities of glucose solution (16.6 mM) or hydrogen peroxide solution (H₂O₂, 1 mM) were dripped on the adhesive interface between hydrogel and porcine skin, respectively, and left at room temperature for 15 minutes to ensure full penetration into the hydrogel network structure and destruction of the cross-linking points. Furthermore, the adhesive interface was treated with the removing agent containing glucose (16.6 mM) and H₂O₂ (1 mM) mixture, which was left for 15 minutes. Thereafter, the residual adhesion force of the hydrogels was determined by universal testing machine at a tensile rate of 10 mm/min. The removal efficiency of the removing agent on the adhesive properties of the hydrogels was calculated by comparing it to the untreated original adhesive tissue. The test was repeated more than three times for each group of hydrogels. The specific calculation formula is as follows:

RR (%) = (1-A_k_/A_c_) × 100%

where A_c_ represents the initial adhesion force of the hydrogel to the wet tissue without any treatment applied, and A_k_ is the residual adhesion force of the hydrogel after the removal agent treatment.

**2.11.2 Burst pressure test**

The injury holes with a diameter of 2 mm were first blocked with hydrogel. Appropriate amounts of glucose solution (16.6 mM) or H_2_O_2_ solution (1 mM) were evenly dripped on the surface of the hydrogel-sealed trauma holes, and left at room temperature for 15 minutes to ensure full penetration and diffusion into the internal network of the hydrogel and the hydrogel-tissue adhesive interface. Furthermore, the adhesive interface was treated with the removing agent containing glucose (16.6 mM) and H_2_O_2_ (1 mM) mixture, which was also left for 15 minutes. The constructed real-time burst pressure monitoring device was then utilized to quantify the residual adhesion of the hydrogel to the tissue after removal agent treatment by injecting PBS into the system at a constant flow rate of 2 mL/min. The adhesion force presented by the hydrogel sealed injury holes was compared with that presented by the untreated hydrogel to calculate the weakening efficiency of the removing agent on the adhesive properties of the hydrogel. The test was repeated more than three times for each group of hydrogels. The calculation method is as follows:

RR (%) = (1-A_k_/A_c_) × 100%

where A_c_ represents the initial adhesion force of the hydrogel to the injury holes without any treatment applied, and A_k_ is the residual adhesion force of the hydrogel after the removal agent treatment.

**2.12 Evaluation of bacterial capture capacity of hydrogels**

**2.12.1 Bacterial capture kinetics of GAPE0U hydrogels**

The target strains were initially inoculated in LB liquid culture medium and subsequently cultured to logarithmic growth stage under suitable conditions. Following the enrichment of bacteria through centrifugation, the bacteria were rinsed three times with saline solution to remove any residual culture medium. After being washed, the bacteria were resuspended and diluted with saline to prepare the bacterial dilution of 10^6^ CFU/mL concentration. Then, 1 mL of bacterial dilution was co-incubated with 300 mg of hydrogel sample at 37°C under constant static conditions. The supernatant was collected at specific time and its absorbance value was measured by enzyme-labeled instrument at 595 nm to reflect the bacterial concentration. Equal volumes of bacterial dilutions without hydrogel were utilized as blank controls and synchronized with the same conditions.^[10]^ Each experimental group was set up with four parallel samples. The bacterial capture rate of the hydrogel was calculated according to the following formula:

BCR (%) = (1-OD_n_/OD_0_) × 100%

where OD_0_ represents the absorbance value of the supernatant of the blank control group and OD_n_ is the absorbance value of the supernatant of the hydrogel treatment group.

**2.12.2 Evaluation of bacterial capture capacity of GAPEU hydrogels**

The GAPEU hydrogels and bacterial dilutions were prepared according to the above procedure. And GAPE0U hydrogel, GAPE1U hydrogel, GAPE2U hydrogel, GAPE3U hydrogel and GAPE4U hydrogel were co-incubated with the bacterial dilution according to the above procedure. The supernatant was collected and its absorbance value was measured by enzyme-labeled instrument to reflect the bacterial concentration. Equal volume of bacterial dilutions without hydrogel was used as the blank control group. Four parallel samples were set for each experimental group. The bacterial capture rate of GAPEU hydrogel was also calculated based on the above formula.

**2.13 Anti-capture test for bacteria**

The bacterial capture performance of the GAPE0U hydrogel was assessed following the aforementioned procedure. Subsequently, bacterial dilutions were collected and subjected to centrifugation to collect bacteria. The collected bacteria were washed five times using saline to remove the interference of hydrogel components for subsequent experiments. The washed bacteria were subsequently re-cultured in LB culture medium, and bacterial dilutions of 10^6^ CFU/mL were configured according to the above procedure. The above steps were repeated 14 times. Assessment of whether bacteria develop capture resistance was done by comparing changes in the capture rate of hydrogels for passaged bacteria. Four parallel samples were set up for each experimental set.

**2.14 In vitro antibacterial testing of hydrogels**

**2.14.1 Bactericidal kinetics of hydrogels**

All hydrogels were irradiated under UV light for 24 hours for subsequent experiments. S. aureus, E. coli, and MRSA were inoculated into liquid culture medium (LB growth medium) and incubated at 37°C in the constant temperature incubator to obtain the bacterial suspension with 10^9^ CFU/mL bacterial concentration. 1 mL of the bacterial suspension was co-incubated with GAPEU hydrogel in a thermostatic shaking chamber (37°C, 150 rpm). The optical density (OD595) value of the bacterial suspension was measured at the specified time. The antibacterial capacity of the hydrogel was calculated according to the following formula. The bacterial suspension without any treatment was utilized as the control group. The experiment was repeated four times for each hydrogel group.

AR (%) = (1-OD_h_/OD_l_) × 100%

where OD_h_ is the optical density value of the bacterial suspension in the GAPEU hydrogel treated group and OD_l_ is the optical density value of the bacterial suspension in the control group.

**2.14.2 Test of antimicrobial capacity of hydrogels by spread plate method**

Sterile GAPEU hydrogels and bacterial suspensions of S. aureus, E. coli, and MRSA (10^9^ CFU/mL) were prepared according to the above conditions. 1 mL of the bacterial suspension and appropriate amount of GAPEU hydrogel were co-incubated in a thermostatic shaking chamber (37°C, 150 rpm) for 12 hours. Subsequently, 50 µL of bacterial incubation solution was diluted, and 50 µL of the diluted bacterial suspension was evenly spread on fresh LB broth agar plates. The plates were then placed in a constant temperature incubator at 37°C. When the bacterial colonies were of appropriate size, they were photographed and counted. The bacterial suspension without any treatment was utilized as the control group for the aforementioned procedure. For each group, the test should be repeated more than three times. The antibacterial capacity of the hydrogel was calculated according to the following formula:

AR (%) = (1-M_h_/M_L_) × 100%

where M_h_ is the number of bacterial colonies on agar plates in the hydrogel treated group and M_L_ is the number of bacterial colonies on agar plates in the group without any treatment.

**2.14.3 Morphological assessment of bacteria after co-incubation with hydrogels**

In order to observe the bacterial morphology intuitively, the bacteria that had been cultured with hydrogel were placed in an environment of 4°C. The fixation treatment was carried out by using glutaraldehyde solution with the concentration of 2.5% for 6 hours. Following fixation, gradient dehydration was conducted with ethanol solutions of 30%, 50%, 70%, 90%, and 100% (v/v), in sequence. Subsequent to the completion of the dehydration process, the bacteria were dried and finally analyzed by field emission scanning electron microscope (JSM-IT800) for bacterial morphology.

**2.15 Drug resistance testing of bacteria**

This approach was based on prior experience and partially modified.^[11]^ The sterile GAPE1U hydrogel and bacterial suspension were prepared according to the above procedure. The appropriate amounts of hydrogel and 1 mL of bacterial suspension were co-incubated for 12 hours. The optical density value of the supernat was tested by the enzyme-labeled instrument to calculate the antibacterial rate of GAPE1U hydrogel. In this case, the bacterial suspension without hydrogel treatment was designated as the control group, and parallel experiments were designed for four groups. The above co-incubated bacterial suspensions were collected and centrifugated to separate the bacteria. The bacterial samples were then washed five times with sterile PBS to ensure the removal of any residual antibacterial agent. Subsequently, the clean bacteria were placed in 1 mL of LB liquid culture medium and cultured to proliferate to 10^9^ CFU/mL. These steps were repeated 14 times. The antibacterial performance of the GAPE1U hydrogel against the passaged bacteria was quantified to assess whether the hydrogel contributed to the development of resistance in the bacteria.

**2.16 Long-term antibacterial performance testing of GAPE3U hydrogel**

The hydrogels and bacterial suspensions were prepared according to the above procedure. 1 mL of the above bacterial suspension was co-incubated with appropriate amount of GAPE3U hydrogel in thermostatic shaking chamber (37°C, 150 rpm), and the optical density value of the bacterial suspension was continuously monitored using the enzyme-labeled instrument. Bacterial suspension without any treatment was used as control group for the above procedure. The test was repeated more than three times for each group. The long-term antibacterial properties of GAPEU hydrogel were evaluated according to the following formula:

AR (%) = (1-OD_h_/OD_l_) × 100%

where OD_h_ is the optical density value of the bacterial suspension in the GAPEU hydrogel treated group and OD_l_ is the optical density value of the bacterial suspension in the control group.

**2.17 Repeated bactericidal performance testing of GAPE3U hydrogel**

According to the above procedure, the GAPE3U hydrogel was co-cultured with S. aureus, E. coli, and MRSA, respectively, for 12 hours. Then, 100 µL of the bacterial suspension was obtained to monitor the bacterial concentration by enzyme-labeled instrument. Bacterial suspensions without any treatment were used as control group for the above procedure. The corresponding antibacterial rate of the hydrogel was calculated according to the above formula. The GAPE3U hydrogel was then removed and rinsed with sterile PBS to eliminate bacteria retained on the surface. And it was put into fresh bacterial suspension (1 mL, 10^9^ CFU/mL) and co-incubated again for 12 hours. The above procedure was repeated three times to evaluate the repeated bactericidal performance of GAPEU hydrogel. The test was repeated more than three times for each group.

**2.18 Testing of hydrogels for biofilm resistance**

Initially, bacterial culture solutions of E. coli, S. aureus and MRSA were placed in 24-well plates and incubated continuously at 37°C under static conditions, and the LB culture solution was changed every 24 h to form mature biofilms. The original culture solution was then replaced with fresh LB culture solution without or with hydrogel samples, which continued to be incubated statically at 37°C to assess the antibiofilm ability of GAPEU hydrogels. The supernatant was carefully pipetted out and the residual biofilm at the bottom of the well plate was lightly washed with PBS and colored with crystal violet. The complete stained biofilm was air-dried and photographed with the digital camera. Afterwards, the binding crystal violet was solubilized with 95% ethanol and the optical density at 562 nm was measured by enzyme-labeled instrument to assess the residual biofilm, thus quantifying the antibiofilm properties of the hydrogels.^[12]^ Each group of tests was repeated four times.

**2.19 Hemocompatibility testing of hydrogels**

Rat arterial blood was firstly collected into the anticoagulation tubes and lyophilized hydrogel was synchronously placed in PBS to completely swell in preparation for the hemocompatibility test. Whole blood samples were centrifuged at 1500 rpm for 10 minutes to separate plasma and RBCs. The lower layer of blood cells was collected and washed several times with PBS to prepare a 4 % erythrocyte suspension. 1 mL of erythrocyte dilution was incubated with the fully swollen hydrogel in a thermostatic shaking chamber at 37°C for 2 hours. Ultrapure water-treated and PBS-treated groups were designated as the positive control and negative control groups, respectively. After incubation, the samples were centrifuged at 1500 rpm for 10 minutes to separate the erythrocyte layer and photographed for recording. 100 μL of supernatant was pipetted into a 96-well plate, and the absorbance value at 540 nm was measured using the enzyme-labeled instrument. Each experimental group was set up with 4 parallel samples. The hemolysis rate was calculated as follows:

HR (%) =(OD_sample_-OD_negative_)/(OD_positive_-OD_negative_) × 100%

where OD_sample_ is the absorbance of the supernatant of the hydrogel-treated group, OD_negative_ is the absorbance of the negative control group (PBS-treated group), and OD_positive_ is the absorbance of the positive control group (ultrapure water-treated group).

**2.20 Cytocompatibility testing of hydrogels**

Experiments to evaluate the cytotoxocity were carried out using the leaching liquor method.^[13]^ The hydrogel leaching solution was prepared by placing sterile hydrogel samples in DMEM complete culture medium containing 10% fetal bovine serum (FBS) and 1% penicillin-streptomycin double antibiotic. L929 or HUVEC cells were then inoculated in well plates at a density of 1.5 × 10^4^ cells/well. Following the cell adhesion to the wall for 24 hours, the original culture medium was discarded. The experimental group was treated with the hydrogel leaching solution, while the control group was replaced with fresh DMEM complete culture medium. The experiment continued until the predetermined time point. The Calcein-AM/PI fluorescent staining kit was utilized to stain live and dead cells, and the cell morphology and proliferation status were observed and evaluated by inverted fluorescence microscopy. Each group was set up with five parallel experiments.

To further quantify cell proliferation, cell inoculation density and culture conditions were consistent with those described above. The steps of changing liquid treatment were consistent with the previous method, and the appropriate amount of CCK-8 reagent was added to each well at the designated time points for staining. The absorbances of each well were subsequently measured at 450 nm using an enzyme-labeled instrument. Each group comprised five parallel experiments. The relative cell activity was calculated according to the following formula:

CV (%) = (OD_sample_-OD_blank_)/ (OD_control_ -OD_blank_) × 100%

where OD_sample_ is the absorbance of the hydrogel leach solution group, OD_control_ is the absorbance of the control group, and OD_blank_ is the absorbance of the CCK-8 working solution without cells.

**2.21 In vivo hemostatic performance assessment of hydrogels**

All the animal experiments involved in this work are approved by the Ethics Committee of the First Affiliated Hospital of Naval Medical University (approval no. CHEC2025-017). Female rats, eight weeks of age, that had adapted to the environment were divided into four groups of four rats each according to the principle of randomization. Subsequent to the administration of anesthesia, the rats were fixed in a supine position on the experimental table. The rats' abdomens were dehaired and subsequently sterilized, and the abdominal cavity was incised layer by layer, fully exposing the liver tissue. A linear wound of approximately 10 mm in length was created on the liver surface. After allowing the wound to bleed naturally for 10 seconds, gauze, medical gelatin sponge, and GAPE3U hydrogel were utilized to stop the bleeding, respectively.^[14]^ The control group received no treatment. Throughout the operation, the hemostasis time and blood loss were meticulously documented.

**2.22 In vitro cell migration assay and in vitro cell tube formation assay**

**2.22.1 In vitro cell migration assay**

First, HUVEC were seeded in 24-well plates. When the cell confluency reached 90%, linear scratches were made on the monolayer cells with a sterile pipette tip, followed by washing with PBS. Subsequently, the cells were grouped and added the corresponding culture medium: control group (pure culture medium), H₂O₂ group (culture medium containing H₂O₂), 3M hydrogel group (culture medium containing H₂O₂ and 3M hydrogel), and GAPE3U hydrogel (culture medium containing H₂O₂ and GAPE3U hydrogel). Cellular migration images of each treatment group were captured with optical microscope at 0 and 12 hours after adding the culture medium. The cell migration areas were quantified using Image J software. Three parallel experiments were set up for each treatment group.

**2.22.2 In vitro cell tube formation assay**

Add 10 μL of matrigel to 96-well plates. After solidification at 37℃, HUVEC were seeded onto the surface of the gel. According to the experimental design, the cells were divided into four groups and added the corresponding culture medium: control group (pure culture medium), H₂O₂ group (culture medium containing H₂O₂), 3M hydrogel group (culture medium containing H₂O₂ and 3M hydrogel), and GAPE3U hydrogel (culture medium containing H₂O₂ and GAPE3U hydrogel). After 12 hours of incubation, the cells were stained with Calcein-AM. Subsequently, images of cell tube formation were captured using a microscope and analyzed with Image J software. Three parallel experiments were set up for each treatment group.

**2.23 Evaluation of wound healing efficacy of hydrogel in infection models of sports skin defects**

All the animal experiments involved in this work are approved by the Ethics Committee of the First Affiliated Hospital of Naval Medical University (approval no. CHEC2025-017). Healthy 6-week-aged C57 male mice were selected and acclimatized for 14 days in an SPF-grade animal house. They were randomly divided into five experimental groups: a) no infected + PBS-treated wounds group, b) infected + PBS-treated wounds group, c) infected + 3M commercialized hydrogel-treated wounds group, d) infected + GAPE2U hydrogel-treated wounds group, and e) infected + GAPE3U hydrogel-treated wounds group. The mice were initially anesthetized, and following the disappearance of the corneal reflex, an electric shaver was utilized to remove the hair at the nape. The disposable skin biopsy punch with the inner diameter of 10 mm was utilized to obtain the full skin defect wound with a diameter of 10 mm by applying vertical pressure. Subsequently, 200 μL of MRSA solution (concentration of 1×10^9^ CFU/mL) was dripped onto the wound surface with a pipette, and then placed in the thermostatic incubator for 5 hours to promote full bacterial infection of the wound. 200 μL of hydrogel or PBS was given to each group corresponding to the treatment to ensure that the hydrogel was completely adhered to the wounds. On postoperative days 0, 3, 5, 9, 13, and 17, digital cameras were utilized to capture images of the wounds. The ImageJ software was utilized to analyze the wound area, and the healing rate was calculated as follows:

Wound healing (%) = (A_0_-A_n_)/A_0_×100%

where A_0_ is the initial area of the wound on postoperative day 0 and A_n_ is the unhealed area of the wound on day n.

On days 3 and 5 after modeling, wound tissues were excised with a sterile scalpel and placed in 1 mL of sterile LB liquid culture medium. The tissue homogenizer was then utilized to perform homogenization operations on the tissues. 50 μL of diluted tissue homogenate was evenly spread on LB solid culture medium plates and inverted in the constant temperature incubator at 37℃. The colonies were incubated until their morphology was clear, and they were photographed and recorded with a digital camera. The experiment was repeated four times for each group. The in vivo antibacterial capacity of each hydrogel group was calculated according to the following formula:

AR (%) = (1-M_h_/M_L_) × 100%

where M_h_ is the number of bacterial colonies on agar plates in groups a, c, d, and e, and M_L_ is the number of bacterial colonies on agar plates in group b.

**2.24 Histologic and macrophage phenotypic assessment of regenerative infected sports wounds**

On postoperative days 9 and 17, some of the mice were euthanized, and the whole skin tissue from the wounds was collected. Tissue samples were immediately pretreated with tissue fixative. After that, the tissue samples were embedded with paraffin wax, and then they were sliced. The skin slices were stained using H&E staining, Masson trichrome staining, TNF-α immunohistochemical staining and immunofluorescence staining to visualize the inflammatory response infiltration and collagen deposition status of the regenerated tissues. Furthermore, the above skin slices were quantitatively analyzed with ImageJ software. The regenerated tissues were subsequently observed for epidermal regeneration thickness, granulation tissue thickness, degree of inflammatory cell infiltration, macrophage phenotype, and follicle regeneration. The experiment was repeated four times for each group.

**2.25 Assessment of vascular regeneration in regenerated tissue from infected sports wounds**

Tissues were obtained and coarsely processed on postoperative days 9, 13, and 17, according to the above-mentioned procedure. Thereafter, they were embedded with paraffin and then sliced. Immunofluorescence staining with CD31 was used on nascent tissue slices to assess the reconstruction of the vascular network in the regenerated tissues. The experiment was repeated four times for each group.

**2.26 Assessment of marginal stress situation of regenerated tissue from infected sports wounds**

Tissues were sliced on postoperative days 13 and 17 according to the procedure described above. Subsequently, mechanotransduction of the nascent tissues was analyzed visually by YAP immunofluorescence, Fak immunofluorescence, TGF-β1 immunofluorescence, and CTGF immunofluorescence. The phenotype of fibroblasts was labeled with En1 immunofluorescence. Meanwhile, the distribution of myofibroblasts in the nascent tissues was visually analyzed using α-SMA immunofluorescence. The experiments were repeated four times for each group.

**2.27 In vivo host response**

The GAPEU hydrogel samples were sterilized by UV irradiation in the ultraclean table for 24 hours, during which the samples were turned over every 6 hours to ensure uniform sterilization. The in vivo biocompatibility of the hydrogel was evaluated using eight-week-aged male C57 mice. 200 μL of sterilized GAPE3U hydrogel was implanted on the back of mice subcutaneously. Seven days after implantation, the mice were euthanized, and the whole skin tissue from the hydrogel implantation site was excised. Tissue samples were immediately pretreated with tissue fixative. Afterwards, the tissue samples were embedded with paraffin wax and then sliced. Skin slices were stained using H&E staining to assess the inflammatory cascade response of the skin in the implanted area.^[15]^ According to the aforementioned experimental protocol, GAPE3U hydrogel was implanted into male C57 mice via subcutaneous injection. Fourteen days after the implantation procedure was completed, the mice were euthanized. Subsequently, their hearts, livers, spleens, lungs, and kidneys were removed and subjected to paraffin embedding. Subsequently, sectioning was performed on the above embedded organ samples, followed by staining with hematoxylin-eosin (namely H&E staining). The degree of inflammatory infiltration and histological structural characteristics of the organ tissues were analyzed based on the staining results. Parallel experiments were set up as four groups.

**2.28 Statistical analysis**

In this study, all the experimental results were presented in the form of “mean ± standard deviation (mean ± SD)”. deviation. Also, GraphPad Prism 9 software was used throughout the statistical analysis. Differences between multiple groups were analyzed by one-way analysis of variance (ANOVA), followed by Tukey's multiple comparison test. All experiments were repeated at least 3 times (n ≥ 3). When p < 0.05 (*),p < 0.01 (**),p < 0.001 (***)，it was judged to be statistically significant, while p > 0.05 (ns), it was determined that there was no significant difference.

Figure S1. (A) Schematic diagram of the preparation of phenylboronic acid-modified gelatin (Gel-APBA), (B) ^1^H NMR analysis spectrum, (C, D, E) UV analysis spectrum.

Figure S2. (A) Schematic diagram of the preparation of norbornene-modified polyvinyl alcohol (PVA-NB), (B) ^1^H NMR analysis spectrum.

Figure S3. Swelling curves of hydrogels. Data are mean ± standard deviation (n ≥ 3).

Figure S4. Pore size analysis of hydrogels. Data are mean ± standard deviation (n ≥ 3).

Figure S5. (A) Hundred compression curves of GAPE0U hydrogel, (B) Time-stress statistic plots of hundred compression of GAPE0U hydrogel, (C) Static compression of GAPE0U hydrogel, and (D) Static tension of GAPE0U hydrogel. Data are mean ± standard deviation (n ≥ 3).

Figure S6. (A, B, C, D, E) Uniaxial tensile tests of original and self-healed hydrogels, (F) Statistical plot of hydrogel self-healing efficiency, (G) Macroscopic pictures of self-healed hydrogels, (H) Diagram of hydrogel self-healing mechanism. Data are mean ± standard deviation (n ≥ 3).

Figure S7. (A) Rheological testing and macroscopic injectability of GAPE hydrogel, (B) Filling on porcine liver injury site with hydrogel.

Figure S8. Microfluidity performance test of GAPE and GAPEU.

Figure S9. (A) Physical image of lap shear, (B) Burst test monitoring device, (C) Detailed images of hydrogel sealing porcine skin injury holes, and (D) Detailed images of hydrogel sealing small intestinal injury holes.

Figure S10. (A, D) Lap-shear test and quantitative analysis after glucose solution treatment of the bonded interface, (B, E) Lap-shear test and quantitative analysis after H_2_O_2_ solution treatment of the bonded interface, (C, F) Lap-shear test and quantitative analysis after glucose/H_2_O_2_ co-solution treatment of the bonded interface. Data are mean ± standard deviation (n ≥ 3).

Figure S11. Bursting tests to examine the ability of glucose solution (A), H_2_O_2_ solution (B), and glucose/H_2_O_2_ co-solution (C) to detach the hydrogel at injury holes of porcine skins. Data are mean ± standard deviation (n ≥ 3).

Figure S12. Bursting tests to examine the ability of glucose solution (A), H_2_O_2_ solution (B), and glucose/H_2_O_2_ co-solution (C) to detach the hydrogel at injury holes of small intestine. Data are mean ± standard deviation (n ≥ 3).

Figure S13. Quantification of the removal capacity of different treatments on mature biofilms. Data are mean ± standard deviation (n ≥ 3).

Figure S14. Quantification of in vivo antibacterial capacity of hydrogels. Data are mean ± standard deviation (n ≥ 3); one-way analysis of variance (ANOVA); P values: *P < 0.05, **P < 0.01, ***P < 0.001.

Figure S15. H&E staining of skin after subcutaneous implantation of hydrogel (scale bar of overall image: 1 mm, scale bar of enlarged image: 100 μm).

Figure S16. (A) In vitro migration images of HUVECs and (B) cell migration rate statistics. Data are mean ± standard deviation (n ≥ 3); one-way analysis of variance (ANOVA); P values: *P < 0.05, **P < 0.01, ***P < 0.001.

Figure S17. (A) In vitro tube formation images of HUVEC and (B) statistics of the number of nodes. Data are mean ± standard deviation (n ≥ 3); one-way analysis of variance (ANOVA); P values: *P < 0.05, **P < 0.01, ***P < 0.001.

Figure S18. H&E staining and Masson staining of wounds treated with “energy transit station” hydrogel (GAPE3U) on days 9 and 17.

Figure S19. (A) Collagen deposition of wounds, (B) Scar width of wounds, (C) Granulation tissue thickness of wounds, (D) Epidermal thickness of wounds, (E) Hair follicle index of wounds. Data are mean ± standard deviation (n ≥ 3); one-way analysis of variance (ANOVA); P values: *P < 0.05, **P < 0.01, ***P < 0.001.

Figure S20. H&E staining of major organs in mice.

Figure S21. Fak immunofluorescence and quantitative statistics (scale bar :100 μm). Data are mean ± standard deviation (n ≥ 3); one-way analysis of variance (ANOVA); P values: *P < 0.05, **P < 0.01, ***P < 0.001.

Figure S22. TGF-β1 immunofluorescence and quantitative statistics (scale bar :100 μm). Data are mean ± standard deviation (n ≥ 3); one-way analysis of variance (ANOVA); P values: *P < 0.05, **P < 0.01, ***P < 0.001.

Figure S23. CTGF immunofluorescence and quantitative statistics (scale bar :100 μm). Data are mean ± standard deviation (n ≥ 3); one-way analysis of variance (ANOVA); P values: *P < 0.05, **P < 0.01, ***P < 0.001.

**References**

[1]	S. Hua, Y. Zhang, Y. Zhu, X. Fu, L. Meng, L. Zhao, L. Kong, S. Pan, Y. Che, Tunicate cellulose nanocrystals strengthened injectable stretchable hydrogel as multi-responsive enhanced antibacterial wound dressing for promoting diabetic wound healing. *Carbohydrate Polymers* **2024**, *343*, 122426. <https://doi.org/10.1016/j.carbpol.2024.122426>.

[2]	X. Yang, M. Chen, P. Li, Z. Ji, M. Wang, Y. Feng, C. Shi, Fabricating poly(vinyl alcohol)/gelatin composite sponges with high absorbency and water-triggered expansion for noncompressible hemorrhage and wound healing. *Journal of Materials Chemistry B* **2021**, *9*, 1568-1582. <https://doi.org/10.1039/d0tb02480e>.

[3]	Y. Jing, T. Huang, B. Zhao, L. Zhao, N. Zhang, K. Zhang, K. Wang, J. Wang, J. Hua, Q. Tu, A ROS/glucose stimulated-responsive ADSCs-derived exosomes-release hydrogel system for diabetic wound healing. *Chemical Engineering Journal* **2024**, *487*, 150561. <https://doi.org/10.1016/j.cej.2024.150561>.

[4]	L. Zhao, J. Wang, Y. Pan, F. Tan, T. Wang, H. Ran, M. Pang, X. Zou, P. Xu, A. Chen, J. Chen, P. Li, M. Zhou, X. Guo, J. Wang, X. Yu, Y. Peng, Selenium-Albumin Nanoaccelerator Hydrogel Promotes Wound Healing by Antibacterial, Anti-Inflammatory and Antioxidant along with Inhibits Scar Formation via Downregulating CD36. *Advanced Healthcare Materials* **2025**, *14*. <https://doi.org/10.1002/adhm.202500699>.

[5]	Y. Qian, Y. Zheng, J. Jin, X. Wu, K. Xu, M. Dai, Q. Niu, H. Zheng, X. He, J. Shen, Immunoregulation in Diabetic Wound Repair with a Photoenhanced Glycyrrhizic Acid Hydrogel Scaffold. *Advanced Materials* **2022**, *34*, 2200521. <https://doi.org/10.1002/adma.202200521>.

[6]	Y. Zhang, S. Lin, X. Xu, Y. Yao, S. Feng, S. Jiang, Y. Wang, W. He, R. Mo, Programmable hierarchical hydrogel dressing for sequential release of growth factor and DNase to accelerate diabetic wound healing. *Journal of controlled release : official journal of the Controlled Release Society* **2025**, *383*, 113825-113825. <https://doi.org/10.1016/j.jconrel.2025.113825>.

[7]	S. Lu, X. Hu, B. Xu, C. Bai, T. Wang, T. Ma, S. Yi, Effects of different salt ions on the structure and rheological behavior of sulfated cellulose nanocrystal hydrogel. *Food Hydrocolloids* **2024**, *151*, 109799. <https://doi.org/10.1016/j.foodhyd.2024.109799>.

[8]	D. Zhong, Z. Wang, J. Xu, J. Liu, R. Xiao, S. Qu, W. Yang, A strategy for tough and fatigue-resistant hydrogels via loose cross-linking and dense dehydration-induced entanglements. *Nature Communications* **2024**, *15*, 5896. <https://doi.org/10.1038/s41467-024-50364-3>.

[9]	Y. Lv, Y. Xu, S. Zhang, S. Xie, B. Wang, T. Sun, X. Zhang, S. Yao, H. Zhang, L. Wang, L.-N. Wang, Rapidly Photocurable and Strongly Adhesive Hydrogel-Based Sealant with Good Procoagulant Activity for Lethal Hemorrhage Control. *Advanced Functional Materials* **2025**. <https://doi.org/10.1002/adfm.202501904>.

[10]	J. Ren, Y. Qiao, L. Jin, C. Mao, C. Wang, S. Wu, Y. Zheng, Z. Li, Z. Cui, H. Jiang, S. Zhu, X. Liu, A Smart Bacteria-Capture-Killing Vector for Effectively Treating Osteomyelitis Through Synergy Under Microwave Therapy. *Small* **2024**, *20*. <https://doi.org/10.1002/smll.202307406>.

[11]	Y. He, X. Wang, S. He, X. Han, A. Wang, F. Zhang, J. Deng, X. Long, J. Lin, Y. Feng, X. He, Z. Li, J. Li, F. Luo, H. Tan, Enhancing Antibiotic-Resistant Bacterial Infection Therapy: Self-Assembling Gemini Quaternary Ammonium-Functionalized Peptide Nanoassemblies with Multiple Antibacterial Mechanisms. *Acs Nano* **2025**, *19*, 6977-6992. <https://doi.org/10.1021/acsnano.4c14689>.

[12]	L. Zong, R. Teng, H. Zhang, W. Liu, Y. Feng, Z. Lu, Y. Zhou, Z. Fan, M. Li, X. Pu, Ultrasound-Responsive HBD Peptide Hydrogel with Antibiofilm Capability for Fast Diabetic Wound Healing. *Advanced Science* **2024**, *11*. <https://doi.org/10.1002/advs.202406022>.

[13]	Z. Zhou, L. Wang, D. Yang, Q. Li, X. Wang, J. Nie, G. Ma, Acid-Triggered Charge-Switchable Antibacterial Hydrogel for Accelerated Healing of Gastric Mucosal Wounds. *ACS nano* **2025**. <https://doi.org/10.1021/acsnano.5c00426>.

[14]	L. Feng, Q. Peng, L. Miao, C. Cai, F. R. Tay, S. Zhou, Y. Zhang, Z. Liu, X. Wang, Y. Jiao, R. Guo, "Monitor-and-treat" that integrates bacterio-therapeutics and bio-optics for infected wound management. *Bioactive Materials* **2025**, *48*, 118-134. <https://doi.org/10.1016/j.bioactmat.2025.02.001>.

[15]	F. Saeedinejad, F. Alipanah, S. Toro, N. Pereira, D. Ghanbariamin, I. Jozic, T. A. Schmidt, E. Arab-Tehrany, Y. S. Zhang, A. Tamayol, M. Samandari, In Situ-Formed Tissue-Adhesive Macroporous Scaffolds Enhance Cell Infiltration and Tissue Regeneration. *Acta biomaterialia* **2025**. <https://doi.org/10.1016/j.actbio.2025.04.049>.
